# Comparison of Bayesian Coalescent Skyline Plot Models for Inferring Demographic Histories

**DOI:** 10.1093/molbev/msae073

**Published:** 2024-04-17

**Authors:** Ronja J Billenstein, Sebastian Höhna

**Affiliations:** GeoBio-Center, Ludwig-Maximilians-Universität München, Munich 80333, Germany; Department of Earth and Environmental Sciences, Paleontology & Geobiology, Ludwig-Maximilians-Universität München, Munich 80333, Germany; GeoBio-Center, Ludwig-Maximilians-Universität München, Munich 80333, Germany; Department of Earth and Environmental Sciences, Paleontology & Geobiology, Ludwig-Maximilians-Universität München, Munich 80333, Germany

**Keywords:** demographic histories, coalescent, heterochronous samples, RevBayes

## Abstract

Bayesian coalescent skyline plot models are widely used to infer demographic histories. The first (non-Bayesian) coalescent skyline plot model assumed a known genealogy as data, while subsequent models and implementations jointly inferred the genealogy and demographic history from sequence data, including heterochronous samples. Overall, there exist multiple different Bayesian coalescent skyline plot models which mainly differ in two key aspects: (i) how changes in population size are modeled through independent or autocorrelated prior distributions, and (ii) how many change-points in the demographic history are used, where they occur and if the number is pre-specified or inferred. The specific impact of each of these choices on the inferred demographic history is not known because of two reasons: first, not all models are implemented in the same software, and second, each model implementation makes specific choices that the biologist cannot influence. To facilitate a detailed evaluation of Bayesian coalescent skyline plot models, we implemented all currently described models in a flexible design into the software RevBayes. Furthermore, we evaluated models and choices on an empirical dataset of horses supplemented by a small simulation study. We find that estimated demographic histories can be grouped broadly into two groups depending on how change-points in the demographic history are specified (either independent of or at coalescent events). Our simulations suggest that models using change-points at coalescent events produce spurious variation near the present, while most models using independent change-points tend to over-smooth the inferred demographic history.

## Introduction

Coalescent models are widely applied to infer demographic histories from aligned nucleotide sequences of multiple individuals from the same population (Drummond et al. 2005; Ho and Shapiro 2011). In the last decades, considerable advances have been made in inferring demographic histories using coalescent models, amongst others, the Bayesian coalescent skyline plot (Drummond et al. 2005; Ho and Shapiro 2011). Now, many different Bayesian coalescent skyline plot models and types of approaches to choose from exist. The main differences can be categorized into (i) how the population size and its changes over time are modeled, (ii) if samples are contemporaneous (i.e. isochronous) or taken at different timepoints (i.e. heterochronous), (iii) if a single locus or multiple loci are analyzed, and (iv) if demographic inference is performed assuming the genealogy as data or estimating the genealogy jointly. In this study, we use a recently published horse dataset (Vershinina et al. 2021) to explore the empirical impact of model and data choice on demographic inference.

All demographic inferences using coalescent skyline plot models fundamentally rely on the same probability distribution function of the genealogy given the population size parameters (Kingman 1982; Griffiths and Tavaré 1994; Felsenstein et al. 1999). The main difference in Bayesian coalescent skyline plot models for inferring demographies lies in how the population size is modeled over time. The most common approach is to divide time into intervals and then to assume either a constant or a linearly changing population size within each interval.

Different models (i) specify a different number of intervals, (ii) use different approaches to infer the change-points between intervals, and (iii) specify how the population size changes between intervals (e.g. autocorrelated or independent). For example, the first (non-Bayesian) coalescent skyline plot model assumed the population size to be independent between coalescent events (Pybus et al. 2000). This approach produced very noisy estimates of demographic histories. Based on this original idea of coalescent skyline plot models, the generalized skyline plot and Bayesian coalescent skyline plot models were developed (Strimmer and Pybus 2001; Drummond et al. 2005). Most of these models combine several coalescent events into one interval and thus estimate more robust and smooth demographic histories. In Bayesian coalescent skyline plot models, averaging across samples of the genealogy adds to the smoothness of the estimated demographic histories although the actual demographic history used in the model is either a piecewise constant or linear function. Further developments focused on model averaging approaches to not rely on a pre-specified number of intervals (Strimmer and Pybus 2001; Opgen-Rhein et al. 2005; Heled and Drummond 2008), or regularizing/smoothing the population size trajectory through autocorrelation (Minin et al. 2008; Gill et al. 2012; Faulkner et al. 2020). See Ho and Shapiro (2011) for a review, noting that some recent approaches have been published afterwards (e.g. Gill et al. 2012; Faulkner et al. 2020).

The development of different flavors of Bayesian coalescent skyline plot models has provided biologists with many choices, and it is not clear what the empirical impact of these choices is on the qualitative interpretation of the inferred demographic history. Additionally, the different flavors of Bayesian coalescent skyline plot models were implemented in different software, for example, BEAST (Suchard et al. 2018) and R (R Core Team 2022). The implementation in different software packages makes comparison of different models challenging as additional inference components are often different, for example, jointly estimating the genealogy vs. assuming the genealogy as data and the choice of hyperprior distributions. When the genealogy is, for example, used as input data, i.e. topology and coalescent times are fixed, phylogenetic uncertainty from estimating the genealogy is ignored. This can have an impact on the estimated demographic history.

To compare different Bayesian coalescent skyline plot models, we (re-)implemented all currently described Bayesian coalescent skyline plot models into the open-source phylogenetics software RevBayes (Höhna et al. 2016). Additional to the previously described and published models, we developed and implemented a new Bayesian coalescent skyline plot model, called *Skyfish*, using a Poisson prior distribution on the number of population size change events, reversible-jump Markov chain Monte Carlo (RJ-MCMC) to infer the number of population size change events and an autocorrelated process to model population size within each interval (inspired by the Bayesian multiple-change-point method by Opgen-Rhein et al. 2005). We explored in total nine different Bayesian coalescent skyline plot models: a *Constant* model, a *Skyline* model with uncorrelated population size values, a Bayesian skyline plot (*BSP*) model (Drummond et al. 2005), an extended BSP model (*EBSP*, Heled and Drummond 2008), a *Skyride* model (Minin et al. 2008), a *Skygrid* model (Gill et al. 2012), a Gaussian Markov Random Field model (*GMRF*, Faulkner et al. 2020), a Horseshoe Markov Random Field model (*HSMRF*, Faulkner et al. 2020), and our new *Skyfish* model (Opgen-Rhein et al. 2005).

We used a recently published horse dataset from Vershinina et al. (2021) to explore the impact on the inferred demography. First, we find that heterochronous data are more powerful, although this is likely mainly due to the increased data size and possibly also due to the increased number of coalescent events deeper in time. Second, we find that Bayesian coalescent skyline plot models can be divided into two main categories based on how change-points are modeled (i.e. either at coalescent events or independent of coalescent events). Within each category, the inferred demographies are qualitatively equivalent. Third, we find that inferred demographies are qualitatively equivalent regardless whether the genealogy was jointly estimated, assumed to be known, or the uncertainty integrated using a set of genealogies.

We provide tutorials for estimating demographic histories using Bayesian coalescent skyline plot models in RevBayes at https://revbayes.github.io/tutorials/coalescent/.

## New Approaches

In this work, we (re-)implemented all published flavors of Bayesian coalescent skyline plot models into a single software, RevBayes (Höhna et al. 2016). The implementation of Bayesian coalescent skyline plot models in RevBayes is provided through one single distribution: dnCoalescentSkyline. This distribution is designed to be flexible by (i) allowing isochronous and heterochronous data, (ii) allowing any number of intervals, (iii) specifying how many coalescent events are included per interval or providing the change-points directly, and (iv) specifying if the population size is constant or linearly changing within an interval. The per-interval population size and change-points are modeled through different prior distributions.

Our implementation thus cleanly separates the coalescent probability density function from the prior distribution on the demographic history and therefore permits the same choices for different Bayesian coalescent skyline plot models (e.g. whether the population size is constant or linearly changing within an interval). Furthermore, our implementation in RevBayes allows free choice of hyperprior distributions (i.e. prior distributions on parameters of prior distributions) so that different Bayesian coalescent skyline plot models can be compared with the same hyperprior distributions (e.g. the same distribution on the population size at the present). Finally, our new implementation in RevBayes provides the opportunity to either jointly estimate genealogies and population size trajectories or to first estimate genealogies and then estimate population size trajectories from a sample of trees or the *maximum a posteriori* (MAP) tree. Thus, our new implementation allowed us to test and compare the most frequently applied Bayesian coalescent skyline plot models in one software, avoiding any differences introduced due to the specific implementation.

## Results

We applied all nine models to an empirical example of horses to compare the performance between different Bayesian coalescent skyline plot models. First, we analyzed mitochondrial genome sequence data from 36 isochronous samples of horses taken from Vershinina et al. (2021). Second, we used the full dataset of 137 additional heterochronous samples, giving a total of 173 samples. In the first two analyses, we jointly estimated genealogies and population size changes through time. In a third analysis, we used the MAP genealogy (topology and coalescent times) as data, or 10 or 100 samples from the posterior distribution of genealogies from the constant population size coalescent analysis.

**Fig. 1.**
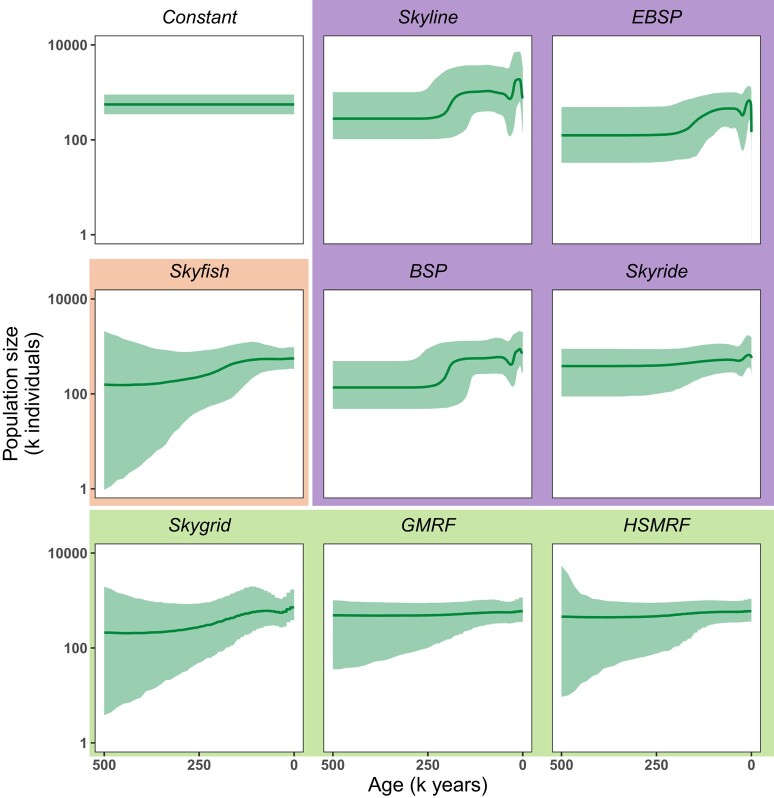
Population size trajectories estimated with nine different models from isochronous sequence data on a timeline from 0 to 500,000 years ago. Genealogies and population size were jointly estimated. All analyses considered possible variation in population size between 0 and 500,000 years ago. The bold line represents the median of the posterior distribution of the population size and the shaded area shows the 95% credible intervals. The four models in the top right corner (*BSP*, *EBSP*, *Skyline*, *Skyride*) are coalescent event based models, i.e. a change in population size can only occur at the time of a coalescent event. In the three models in the bottom row (*Skygrid*, *GMRF*, *HSMRF*), population size changes can happen at specified times, independent from coalescent events. Here, all intervals for these models are equally sized. In the *Skyfish* model, the number of intervals as well as their duration are estimated. All models except for the *Constant* model, the *EBSP* model, and the *Skyline* model have correlated intervals. MCMCs were run for 100,000 iterations, sampling every tenth iteration, with a burn-in of 10% and two replicates, yielding 18,000 samples in total. For plotting, the resulting trajectories were evaluated at 500 exponentially spaced grid points between 0 and 500,000 years ago.

For mitochondrial genome sequence data from 36 isochronous samples, the results vary in various aspects (Fig. 1). The results can primarily be grouped according to how change-points are specified, which we highlight with different background colors in Fig. 1. Models with change-points at coalescent events (highlighted in violet) produced the most variable demographic history. All four models inferred recent changes with a peak in population size very close to the present. Additionally, all models except the *Skyride* model inferred a population size increase about 250 K to 220 K years ago. The *Skyline* model, which does not include autocorrelation compared to the standard *BSP*, inferred the most variation among the coalescent event based models. Moreover, the shape of the prior distribution on the population size influenced the overall demographic history to a smaller population size on average when assuming an exponential distribution (e.g. *BSP* and *EBSP* models) compared to assuming a uniform distribution (e.g. *Skyline* model).

None of the remaining models inferred changes in population size close to the present. The models with pre-specified change-points (Fig. 1 bottom row: Skygrid, GMRF and HSMRF) and the *Skyfish* model inferred a less variable demographic history. The *Skyfish* and the *Skygrid* model inferred a population size increase about 250 K years ago, however, the increase was less steep and more gradual. Overall, models that allow *a priori* more variation (e.g. *Skygrid* vs. *GMRF* and *Skyline* vs. *BSP*) also inferred more variation in the demographic history. Thus, models which reduce variation *a priori* might be overly smoothing.

**Fig. 2.**
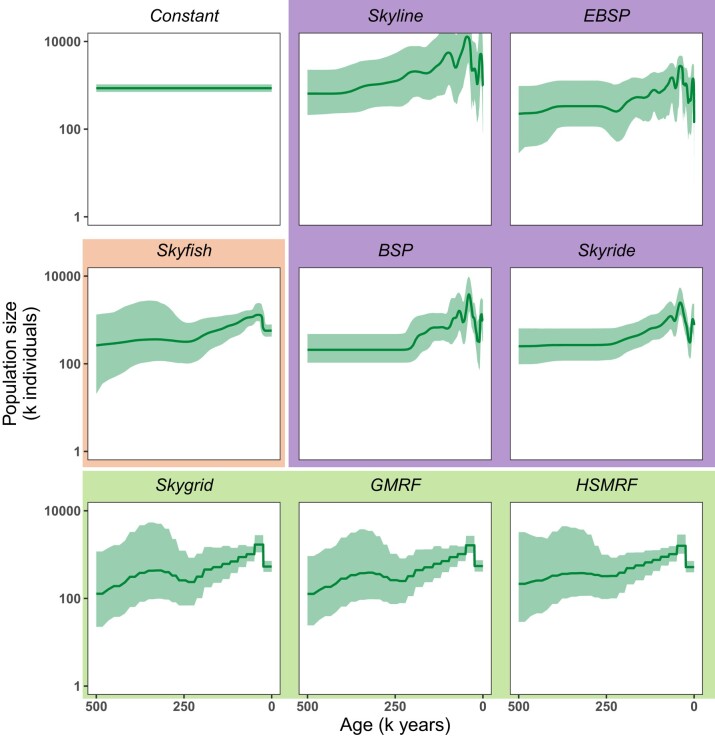
Population size trajectories estimated with nine different models from heterochronous sequence data on a timeline from 0 to 500,000 years ago. Genealogies and population size were jointly estimated. All analyses considered possible variation in population size between 0 and 1,200,000 years ago. The bold line represents the median of the posterior distribution of the population size and the shaded area shows the 95% credible intervals. The four models in the top right corner (*BSP*, *EBSP*, *Skyline*, *Skyride*) are coalescent event based models, i.e. a change in population size can only occur at the time of a coalescent event. In the three models in the bottom row (*Skygrid*, *GMRF*, *HSMRF*), population size changes can happen at specified times, independent from coalescent events. Here, all intervals for these models are equally sized. In the *Skyfish* model, the number of intervals as well as their duration are estimated. All models except for the *Constant* model, the *EBSP* model, and the *Skyline* model have correlated intervals. MCMCs were run for 100,000 iterations, sampling every tenth iteration, with a burn-in of 10% and two replicates, yielding 18,000 samples in total. For plotting, the resulting trajectories were evaluated at 500 exponentially spaced grid points between 0 and 500,000 years ago.

The results of the demographic inference from the heterochronous data can similarly be categorized according to the change-point method (Fig. 2). First, the coalescent event based models (Fig. 2 top right corner: BSP, EBSP, Skyline, Skyride) show the largest amount of population size variation and a peak followed by a decrease near the present. Second, the pre-specified change-point models (Fig. 2 bottom row: Skygrid, GMRF and HSMRF) and the *Skyfish* model (highlighted in orange) inferred smoother demographic histories and corroborate the recent peak followed by a decrease near the present. Third, the latter models also inferred an increase in population size about 600 K years ago, however, the uncertainty, i.e. 95% credible interval, is very large in the time before 600 K years ago (supplementary figure S2, Supplementary Material online).

Our model selection analyses via Bayes factors given the isochronous data supported the *Skyfish* model, and in general supported interval-based change-point methods over change-points at coalescent events (supplementary table S1, Supplementary Material online). The model selection results for the heterochonous data support the *Skygrid* model followed by the *Skyride* model and *HSMRF* model (supplementary table S1, Supplementary Material online). Thus, the model selection results for the heterochronous data show a different ordering of supported models, which might be because we used the same prior distributions, i.e. the same amount of expected variation, although the two analyses use data with different timespans and different amount of samples. Overall, the model selection results show a clear sensitivity to the chosen (hyper-)prior distributions. We note that model selection using Bayes factor can only be performed if proper prior distributions are used (Baele et al. 2013; Baele and Lemey 2014). RevBayes is more strict in specifying proper prior distributions (as initial values are simulated from the specified distribution, e.g. the frequently used 1/x prior distribution is always specified with boundaries) and we have provided the first implementation of the *BSP* with proper prior distributions (see Section *Material and Methods*).

**Fig. 3.**
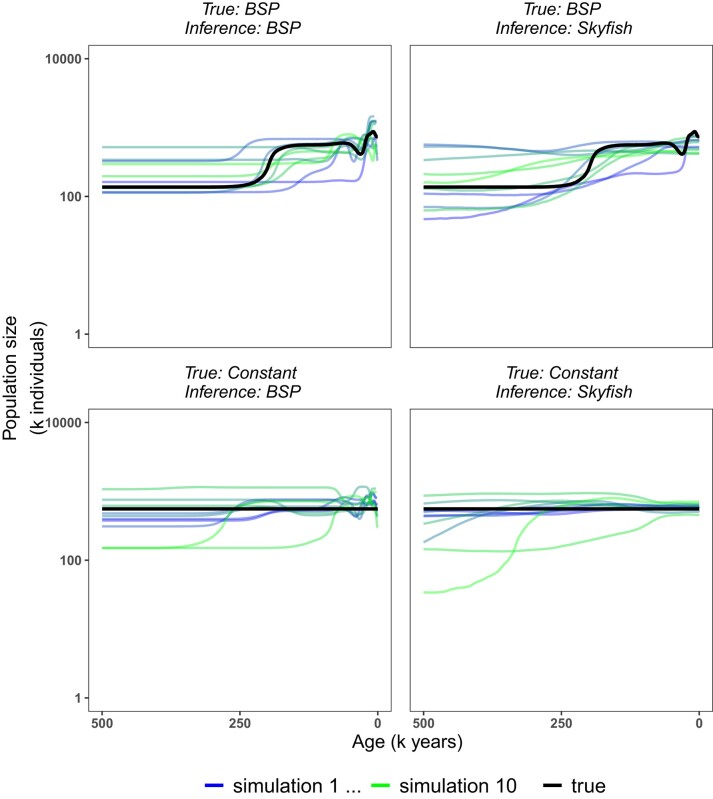
Simulation study results. Ten simulations of sequence data with 36 tips were performed under the resulting population size trajectory from the *BSP* analysis (top row) and the *Constant* analysis (bottom row), both with isochronous data. Analyses of each of the simulations were performed with the *BSP* model (left column) and the *Skyfish* model (right column). The true trajectory used for simulations is depicted in black, medians of the resulting analyses are depicted in hues of blue to green.

Our simulation study supports the results obtained from the empirical data (Fig. 3). The *BSP* model, as a representative for the coalescent event based models, showed a tendency to infer population size changes near the present even when the true demographic history used for simulation was constant. We validated our implementation of the *BSP* by performing the exact same analysis in RevBayes and BEAST to rule out any implementation issues of the likelihood function and the MCMC algorithm (supplementary figure S13, Supplementary Material online). The *Skyfish* model showed a slight tendency to over-smooth the demographic history.

**Fig. 4.**
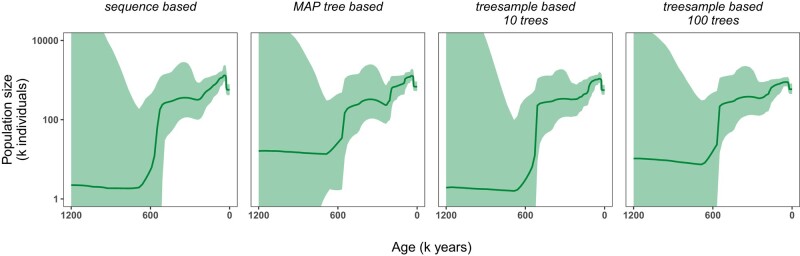
Comparing joint and sequential inference of population size trajectories with the *Skyfish* model from heterochronous data. The *Skyfish* analysis was run between 0 and 1,200,000 years ago. From left to right: sequence-based analysis from the original data; MAP tree based analysis using the MAP tree from the *Constant* analysis with sequence data; analysis based on 10 trees from the posterior distribution of the *Constant* analysis with sequence data; analysis based on 100 trees from the posterior distribution of the *Constant* analysis with sequence data. The bold line represents the median of the posterior distribution of the population size and the shaded area shows the 95% credible intervals.

Our analyses comparing joint inference of the demographic history and genealogy to the inference using either the MAP genealogy or a sample of genealogies from their posterior distribution (all based on an initial analysis assuming a constant size population model) show very similar patterns (Fig. 4). The inferred demographic history is less smooth when only one genealogy (e.g. the MAP genealogy) or few samples are used. There appears to be a small impact of the original demographic history (a constant model) when using the tree samples as data as the magnitude of the population size changes is reduced. Nevertheless, even few samples from the posterior distribution used in a sequential analysis seem to produce qualitatively similar demographic histories, with more samples leading to better matches between the joint and sequential analysis. Lineages through time plots comparing different numbers of genealogies from the posterior distribution of the analysis with the *Constant* model show that there is little variation in the timing of coalescent events, thus corroborating that even few samples from the posterior distribution can be a sufficient approximation compared to a joint inference (supplementary figure S16, Supplementary Material online). We emphasize that the robustness of using some samples compared with the joint inference will be dataset dependent and needs to be explored on a case-by-case basis (Höhna and Hsiang 2024).

We observed that assuming a linearly changing population size compared to a constant population size within each interval produces smoother demographic histories (supplementary figure S9, Supplementary Material online). Nevertheless, the qualitative trends, e.g. peaks, directional changes in population size, and magnitude of population size, are the same regardless whether a constant or linearly changing model was used.

In this study, we newly developed a Bayesian multi-change-point model called *Skyfish* using a Poisson distributed number of change-points. For this model, we explored whether the population size after a change-point should be drawn from an independent prior distribution, an empirically estimated prior distribution, or as an autocorrelated process (supplementary figure S11, Supplementary Material online). Both implementations of uncorrelated population size values produced extremely wide credible intervals and generally do not behave well.

## Discussion

The first coalescent skyline plot models (Pybus et al. 2000), and subsequently the first Bayesian coalescent skyline plot models (Drummond et al. 2005; Heled and Drummond 2008; Minin et al. 2008) used coalescent events to define the change-points in population size. This idea was motivated by reducing the number of parameters as change-points did not have to be estimated separately. The alternative approach is either to estimate the number of change-points together with their position (the multiple-change-points model, Opgen-Rhein et al. 2005) using, for example, RJ-MCMC, or to use regularizing and smoothing priors such as the GMRF (Gill et al. 2012; Faulkner et al. 2020) and its extension, the Horseshoe Markov Random Field (HSMRF, Faulkner et al. 2020). In our empirical comparison, we observed that this distinction—how change-points are modeled—constitutes to the strongest impact on the inferred demographic history.

Choosing the number of intervals in Bayesian coalescent skyline plot models has motivated much of the model development. On the one hand, a larger number of intervals is beneficial to be able to capture more variation in the underlying demographic history. On the other hand, a larger number of intervals comes with additional parameters and model complexity which increases uncertainty (primarily in Bayesian analyses) and can overfit (primarily in Maximum-Likelihood analyses), thus reducing parameter estimation accuracy. For the *BSP* model, it is still necessary to specify the number of intervals. In our example, we used an average of five coalescent events per interval as this provides still some information per interval while giving enough flexibility to model population size variation (Drummond et al. 2005). The *EBSP* model circumvents this problem by applying a Bayesian stochastic variable search algorithm (or RJ-MCMC as in our implementation) and should therefore be preferable over the *BSP* model. In principle, the *Skygrid*, *GMRF*, and *HSMRF* can be used with any large number of intervals (Magee et al. 2020). The global smoothing parameter can be adjusted so that the same total variation is expected *a priori* regardless of the number of intervals. Thus, it is advisable to use the largest number of intervals that improves the smoothness of the inferred demographic history and is computationally feasible (Magee et al. (2020) suggested 100).

Here we argue that coalescent event independent change-point models (*Skygrid*, *GRMF*, *HSMRF*, and *Skyfish*) are superior to coalescent event based models (*Skyline*, *BSP*, *EBSP*, and *Skyride*). First, in our simulation study (Fig. 3), we observed that the *BSP* model, as a representative for all coalescent event based models, inferred spurious population size changes near the present. Our validation comparing the exact same *BSP* analysis in BEAST and RevBayes ruled out that this spurious result is due to our specific implementation (supplementary figure S13, Supplementary Material online). Second, coalescent event based models, with the exception of the *EBSP* (Heled and Drummond 2008), cannot be used for multi-locus datasets since the coalescent events are different for gene trees from different loci and thus the induced demographic history is different. Coalescent event independent change-point models do not share this restriction and can easily be applied to multi-locus datasets as the demographic history is independent of the specific gene tree. Third, coalescent event based models are intrinsically inconsistent when used for simulations. The simulated demographic history depends on the realized coalescent events, thus different simulations with the same vector of population size values have different demographic histories. Similarly, one could see in a graphical model representation (Höhna et al. 2014) that the probability of the genealogy (the coalescent probability) depends on the demographic history, which is constituted by the population size parameters and change-points, while the change-points, and therefore also the demographic history, depend on the genealogy. This reciprocal dependency clearly violates the acyclic condition of well formulated probabilistic models.

In our empirical comparison (Fig. 1) and simulation study (Fig. 3), we observed that some models (e.g. the *GMRF* and *HSMRF*) over-smooth, i.e. are too conservative by favoring unduly a constant population size demography. The expected variation in the *GMRF* and *HSMRF* models is regulated through the standard deviation parameter, which is assigned a hyperprior distribution. The *Skygrid* model, which is equivalent to the *GMRF* model except this hyperprior distribution, is less harsh in penalizing variation (Fig. 1). It might be that the original suggestion for the hyperprior distributions of the *GMRF* and *HSMRF* parameters (Faulkner et al. 2020; Magee et al. 2020) are too conservative and should be revised.

Furthermore, we found no qualitative differences in the inferred demographic history whether we used a linearly changing or a constant population size within an interval (supplementary figure S9, Supplementary Material online). Our comparison between linearly changing and constant population size values within intervals used the *Skyfish* model. In models with a fixed number of intervals, the distinction between linearly changing and constant per-interval population size values will decrease with an increasing number of intervals, while it can be exacerbated for few intervals. We recommend to use the *GMRF* or *HSMRF* model with 50 or more intervals (see also Magee et al. 2020) and therefore it should be less important whether to choose a linearly changing or a constant per-interval population size.

Similarly, we found no qualitative differences in the inferred demographic history whether we jointly inferred the demographic history and genealogy or assumed the genealogy to be known (Fig. 4). Using the genealogy as data has the clear advantage that multiple Bayesian coalescent skyline plot models can be compared efficiently. In theory, joint inference should be superior to sequential inference especially if the genealogy including the coalescent times are impacted by the demographic model (Höhna and Hsiang 2024). However, if the data are sufficiently informative, then the genealogy including the coalescent times are robustly inferred regardless of the chosen demographic model (see supplementary figure S16, Supplementary Material online), e.g. a constant population size model compared with a *Skyfish* Bayesian coalescent skyline plot model.

The *Skyfish* model assumes a Poisson prior distribution for the number of change-points. In our study, the posterior distribution of the number of change-points differs slightly from the prior distribution (see supplementary figure S15, Supplementary Material online). It is, however, difficult to interpret this as a final number of change-points as the population size is correlated between neighboring intervals. This results in a reduced strength of changes and counteracts an increase in change-points during the MCMC.

We noticed that both implementations of the *Skyfish* model with uncorrelated population size values produced extremely wide credible intervals and generally do not behave well (supplementary figure S11, Supplementary Material online). Specifically in the older portion of the process where there are fewer coalescent events and thus little to no information which causes the process to sample solely from the prior distribution. Note that the likelihood function is identical between all the models, thus the poor performance is only due to the choice of prior model. The autocorrelated process clearly outperforms the uncorrelated prior models.

In this study, we only considered single-locus analyses as the horse dataset (Vershinina et al. 2021) consists of mitochondrial DNA. Nevertheless, our implementation also allows the use of multi-loci datasets, if the coalescent event independent change-point models (*Skygrid*, *GRMF*, *HSMRF*, and *Skyfish*) are used. Using multiple loci provides the advantage that each genealogy represents an independent realization of the coalescent process and thus increases more the information in the data compared to adding more sampled individuals.

The Bayesian coalescent skyline plot models described in this study all assume a single panmictic population with data from one locus or multiple loci well representing the population. Recent studies have considered different aspects potentially influencing the accuracy of these models, such as ancient DNA damage or population structure (e.g. Rambaut et al. 2008; Heller et al. 2012, 2013). We recommend to thoroughly examine the data to potential violations of the underlying model assumptions.

Our new implementation of Bayesian coalescent skyline plot models in RevBayes can take full advantage of the flexibility of RevBayes. For example, the original *BSP* model uses autocorrelated exponential distributions while in RevBayes this choice can easily be changed to any other distribution (e.g. autocorrelated normal or lognormal distributions). Similarly, the original *EBSP* model uses an independent exponential distribution which could easily be changed in RevBayes to use any autocorrelated model. Furthermore, our new implementation of Bayesian coalescent skyline plot models allows any of the models to be used with (i) isochronous or heterochronous data, (ii) a linearly changing or a constant population size per interval, and (iii) performing joint inference of the demographic history and the genealogy or assuming the genealogy to be known. Thus, our new implementation facilitates both easier new model development and model comparison among Bayesian coalescent skyline plot models.

## Materials and Methods

### The Coalescent Process

**Fig. 5.**
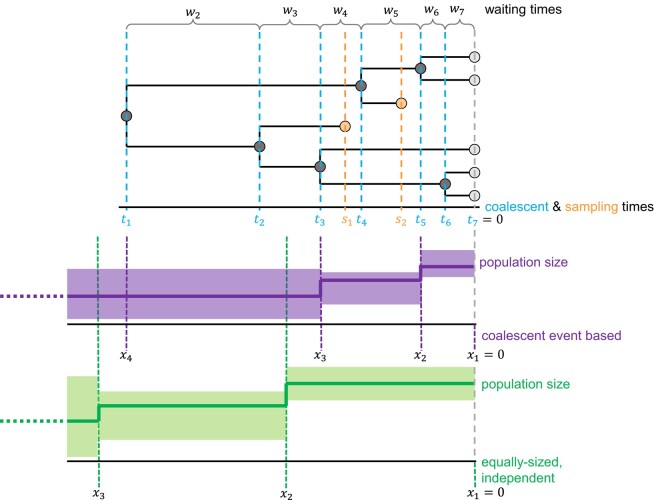
Schematic figure of a coalescent tree with seven samples. Time runs backwards, from samples to the most recent common ancestor (right to left). Coalescent events t are marked by dashed blue lines. Waiting times $w_{m}$ between coalescent events start at time $t_{m}$ and end at time $t_{m-1}$. Sampling times s are marked by dashed orange lines. Below the genealogy, two realizations of a Bayesian coalescent skyline plot model with a constant population size within intervals are shown. In violet: coalescent event based model, the interval change-points coincide with coalescent events. In green: model with interval change-points independent from coalescent events, with equally sized intervals. The solid horizontal lines show the median population size in the intervals, the shaded areas are their credible intervals. Change-points are denoted by *x* on the timeline.

The coalescent process is a flexible, albeit simple, model to represent the process of ancestry among a sample of individuals (Kingman 1982). Here, we use the standard Kingman’s coalescent which assumes a panmictic population of *n* haploid individuals and an effective population size $N_{e}(t)$ at time *t*. Specifically, we assume a *heterochronously* sampled coalescent process where samples can be taken at any time in the past, and our equations collapse to the standard *isochronous* coalescent if all samples are taken at the present time. The following equations and methods can easily be adopted to diploid (or any ploidy levels) by multiplying the effective population size with 2 (or corresponding ploidy levels). Following standard practice, we let time *t* go backwards and start at the present with t=0 for convenience. The coalescent process randomly picks two lineages to coalesce, i.e. merge together, with the waiting times *w* between coalescent events *t* being exponentially distributed (see Fig. 5 for a schematic). In this work, we refer to coalescent time $t_{m}$ as the time of the $(n-m{)}^{th}$ coalescent event (Fig. 5). $t_{n-1}$ thus refers to the most recent coalescent event and $t_{1}$ to the event coalescing the last two lineages.

The coalescent rate at a specific point in time, *t*, is defined by the number of current lineages *k* and the underlying effective population size $N_{e}$:


(1) $$c(t)=\frac{k(t)(k(t)-1)}{2N_{e}(t)}.$$

In case of a coalescent event, the number of active lineages *k* is decreased by 1, a sampling event increases *k* by 1.

Let $u=\{u_{1}=0,u_{2},\ldots ,u_{r}\}$ be the ordered times of all *r* events, including interval change-points x, coalescent events t, and sampling events s (see Fig. 5). The probability of observing the coalescent times t, and thus the genealogy, is calculated by:


(2) $$\begin{array}{rl} P(t|N_{e}(t)) & =\left(\prod_{2<j<r}exp\left[-\int_{u_{\;j-1}}^{u_{j}}c(t)dt\right]\right)\\ & \times \left(\prod_{2<j<r}\frac{2}{k(t)(k(t)-1)}c(u_{j}{)}^{\delta_{j}}\right), \end{array}$$

with $\delta_{j}=1$ if $u_{j}$ is a coalescent event and 0 otherwise. The first term is the probability of no coalescent event between events $u_{\;j-1}$ and $u_{j}$, the second term represents the probability of a coalescent event happening at time $u_{j}$. If δ=0, the second term reduces to 1.

Note that the coalescent probability density function uses the coalescent times in the same units as the genealogy. Thus, if the genealogy is inferred in units of generations, for example if the mutation rate *μ* is known, some external time calibration are used or heterochronous sequence data are used, then our framework directly estimates the effective population size $N_{e}$ instead of the compound parameter $\theta =2N_{e}\mu$.

#### Interval Change-Points

It is common practice to divide time into intervals (Fig. 5) instead of using any continuously varying demographic function. Breaking time into intervals is primarily for mathematical and computational convenience so that equation (2), specifically the integral, can be computed analytically. Subsequently, much work has been done to develop approaches for how to model *interval change-points*. Broadly speaking, there are two main approaches to define the interval change-points (Fig. 5): (i) interval change-points co-occur exactly at coalescent times, and (ii) interval change-points are independent of coalescent events.

Approaches that use coalescent event based interval change-points need to consider (i) how many intervals to choose, and (ii) how many coalescent events occur within an interval (especially if this number is assumed to vary between intervals). The number of intervals can be specified *a priori* or be inferred, e.g. using methods such as Bayesian Stochastic Search Variable Selection (George and McCulloch 1993) or RJ-MCMC sampling (Green 1995). In general, a larger number of intervals has the advantage to be more flexible to capture highly variable demographic histories while too many intervals can lead to noisy, uncertain or even biased estimates.

Independent interval change-points can, in principle, be distributed in any way over the timeline. However, the most common approach uses equally sized intervals (Gill et al. 2012; Faulkner et al. 2020). Note that equally sized intervals generally refers to equal sizes in actual time even though time is sometimes visualized on a logarithmic scale (e.g. Li and Durbin 2011; Liu and Fu 2020). In these models, the number of intervals is fixed *a priori* and chosen large enough to capture the variation in the underlying demography but small enough to remain computationally feasible, e.g. using 50–200 intervals. RevBayes provides the option to place interval change-points either on coalescent events or independently.

#### Demographic Functions within Intervals

There exist two common approaches to modeling the population size within an interval in Bayesian coalescent skyline plot models. We parameterize both approaches the same way by giving a vector *L* of population size values *ρ*, where *L* is equal to the number of intervals. First, a constant population size per interval is assumed. That is, the population size $N_{e}(t)$ is given by


(3) $$N_{e}(t)=\left\{ \begin{array}{cc} \rho_{i}, & \text{if}\;x_{i+1}>t>x_{i},L>i\\ \rho_{L}, & \text{if}\;t>x_{L}, \end{array}\right.$$

with *x* being the interval change-points (see Fig. 5). Second, the population size within an interval is assumed to change linearly. We compute the slope by


(4) $$\alpha_{i}=\frac{\rho_{i+1}-\rho_{i}}{x_{i+1}-x_{i}}.$$

Then, the population size at any time is computed by


(5) $$N_{e}(t)=\left\{ \begin{array}{cc} \rho_{i}+(t-x_{i})\;*\;\alpha_{i}, & \text{if}\;x_{L}>t>x_{i}\\ \rho_{L}, & \text{if}\;t>x_{L}. \end{array}\right.$$

For both approaches, constant and linearly changing per-interval population size, the integral in equation (2) can easily be computed and thus an analytical solution to the likelihood function is known.

In most previous approaches, only the constant per-interval population size model was implemented and used. In our implementation in RevBayes, we provide both options. Since our implementation of any Bayesian coalescent skyline plot model requires a vector of population size values, we can fully support constant and linearly changing per-interval population size.

### Bayesian Coalescent Skyline Plot Models

The main difference between Bayesian coalescent skyline plot models, besides which change-point approach to use, is how the hyperprior distribution on the population size parameters is specified. Here, we present nine different models from the published literature that we implemented in RevBayes and used in this study (see Table 1). Through the presentation of these models together with our comments it should become clear how these models are related to another and could be changed, e.g. by replacing specific hyperprior distributions. The simplicity of exploring alternative models is facilitated through our flexible implementation. The hyperprior distributions presented here are specific choices for the empirical dataset used in this study. They are chosen based on results from Vershinina et al. (2021) and are conservative, i.e. spanning a large range of possible values.

**Table 1:** Overview of all Bayesian coalescent skyline plot models in this paper

Model	Interval change-point method	Autocorrelation	Multiple loci analysis possible?
Constant	—	—	Yes
Skyline	coalescent event based	No	No, only single loci
Bayesian Skyline Plot (BSP) (Drummond et al. 2005)	coalescent event based	Yes	No, only single loci
Extended Bayesian Skyline Plot (EBSP) (Heled and Drummond 2008)	coalescent event based, number and position estimated	No	Yes, but only in BEAST
Skyride (Minin et al. 2008)	coalescent event based	Yes	No, only single loci
Skygrid (Gill et al. 2012)	independent, equally sized	Yes	Yes
Gaussian Markov Random Field (GMRF) Prior (Faulkner et al. 2020)	independent, here equally sized	Yes	Yes
Horseshoe Markov Random Field (HSMRF) Prior (Faulkner et al. 2020)	independent, here equally sized	Yes	Yes
Skyfish (similar to Opgen-Rhein et al. (2005))	independent, number and position estimated	Yes	Yes

####  

##### Constant

The simplest Bayesian coalescent model is to assume a single constant population size,


(6) ρ∼Uniform(0,1E8).

This model does not infer any changes in population size and is included solely as a reference model.

##### Skyline

Our *Skyline* model is equivalent to a Bayesian version of the original coalescent skyline plot (Pybus et al. 2000). This *Skyline* model is not widely used but included here for its simplicity. The *Skyline* model assumes that each population size per interval is independently and identically distributed (uncorrelated),


(7) $$\rho_{i}\;∼\;\text{Uniform}(0,1E8).$$

Here we assumed a uniform prior distribution on the uncorrelated population size, but any other distribution, such as the log-uniform, lognormal, or gamma distribution could be used instead. These models could include fixed or hierarchical models where the hyperparameter, e.g. the mean of the prior distribution is estimated. Our *Skyline* model assumes that intervals change at coalescent events. We arbitrarily decided to merge five coalescent events into one interval, thus, every fifth coalescent event corresponds to an interval change-point.

##### BSP

The widely used *Bayesian Skyline Plot* (BSP) model (Drummond et al. 2005) assumes autocorrelated, exponentially distributed population size values,


(8) $$\begin{array}{rl} \rho_{1} & ∼\text{Loguniform}(1E-2,1E8)\\ \rho_{i+1} & ∼\text{Exponential}(1/\rho_{i}). \end{array}$$

The exponential distribution to model autocorrelation might appear as an unconventional choice and other models, such as normal and lognormal distributions to model autocorrelation are possible.

The *BSP* model assumes that interval change-points occur at coalescent events. Moreover, the *BSP* model (implicitly) assumes a multinomial distribution for the number of coalescent events per interval with at least one event per interval although this distribution is neglected in BEAST and instead an improper prior used (Baele and Lemey 2014). In our analyses, we specified the number of events as $k=\lceil \frac{\left(n-1\right)}{5}\rceil$, i.e. expecting on average five coalescent events per interval. supplementary figure S17, Supplementary Material online shows the graphical model of the *BSP* model.

##### EBSP

The *Extended Bayesian Skyline Plot* (EBSP, Heled and Drummond 2008), primarily developed for multi-locus datasets, assumes that population size values per interval are either independently and exponentially distributed or equivalent to the population size in the previous interval,


(9) $$\rho_{i}\;∼\left\{ \begin{array}{c} \text{Exponential}(\lambda )\\ \rho_{i-1}. \end{array}\right.$$

In the original implementation in BEAST, a Bayesian Stochastic Search Variable Selection algorithm (George and McCulloch 1993) switches between the options if a population size parameter $\rho_{i}$ is equivalent to its predecessor or not. The total number of intervals is distributed according to a truncated Poisson distribution (Heled and Drummond 2008). In our implementation in RevBayes, we use an RJ-MCMC algorithm (Green 1995). Specifically, we specify *a priori* a probability *α* if the population size parameter $\rho_{i}$ is equivalent to its predecessor and apply an RJ-MCMC move that switches between either using the previous population size value or draws a new value from an exponential distribution (for more details, see Freyman and Höhna 2018). The total number of intervals is then binomially distributed with parameters *k* and *α*.

##### Skyride

The *Skyride* model (Minin et al. 2008) assumes autocorrelated population size values which are normally distributed centered on the population size value of the previous interval,


(10) $$\begin{array}{rl} \tau & ∼\text{Gamma}(0.001,0.001)\\ \rho_{1} & ∼\text{Loguniform}(1E-2,1E8)\\ \rho_{i+1} & ∼\text{Lognormal}\left(\rho_{i},\frac{1}{\sqrt{\tau }}\right). \end{array}$$

The *Skyride* model assumes one interval per coalescent event. The large number of intervals is feasible due to smoothing that is governed by the hyperparameter *τ* (Minin et al. 2008). In our implementation, the standard deviations of the normal distributions do not depend on the length of the previous interval, which would make the model resemble a time-dependent Brownian motion (Minin et al. 2008), because the graphical model design in RevBayes prohibits circular dependencies of realizations (the coalescent events) and parameters (the standard deviation) (Höhna et al. 2014).

##### Skygrid

The *Skygrid* model (Gill et al. 2012), similar to the *Skyride* model, assumes autocorrelated population size values which are normally distributed,


(11) $$\begin{array}{rl} \tau & ∼\text{Gamma}(0.001,0.001)\\ \rho_{1} & ∼\text{Loguniform}(1E-2,1E8)\\ \rho_{i+1} & ∼\text{Lognormal}\left(\rho_{i},\frac{1}{\sqrt{\tau }}\right). \end{array}$$

In fact, the prior distributions on the population size values of the *Skygrid* model (equation (11)) are identical to the prior distributions of the *Skyride* model (equation (10)). The only difference between the two models is that interval change-points are independent of coalescent events for the *Skygrid* model and commonly equally distributed among a pre-specified range.

##### GMRF

The *Gaussian Markov Random Field* (GMRF, Faulkner et al. 2020) model assumes autocorrelated population size values which are normally distributed on a logarithmic scale,


(12) $$\begin{array}{rl} \zeta & =0.0195\\ \gamma & ∼\text{Half-Cauchy}(0.0,1.0)\\ \rho_{1} & ∼\text{Uniform}(0,1E8)\\ \ln\;(\rho_{i+1}) & ∼\text{Normal}(\ln\;(\rho_{i}),\gamma \zeta ). \end{array}$$

The GMRF model is, in principle, identical to the *Skygrid* model (equation (11)). The primary difference is how the smoothing parameters *τ* (in the *Skygrid* model) and γζ (in the *GMRF* model) are defined. The *Skygrid* model has a somewhat arbitrary Gamma prior distribution with mean 1.0 and variance 1,000.0. The *GMRF* model, on the other hand, has a standard half-Cauchy prior distribution, which actually does not have a well defined mean and variance. Nevertheless, the smoothing parameter *ζ* is chosen so that the expected variance of the first to last interval is one order of magnitude (Magee et al. 2020). Thus, the value *ζ* is crucial to specify the amount of *a priori* expected variation in the demographic history and depends on the number of intervals. Corresponding *ζ* values can be computed in the R package RevGadgets (Tribble et al. 2022).

##### HSMRF

The *Horseshoe Markov Random Field* (HSMRF, Faulkner et al. 2020) model assumes autocorrelated population size values which are normally distributed on a logarithmic scale,


(13) $$\begin{array}{rl} \zeta & =0.0051\\ \gamma & ∼\text{Half-Cauchy}(0.0,1.0)\\ \rho_{1} & ∼\text{Uniform}(0,1E8)\\ \sigma_{i} & ∼\text{Half-Cauchy}(0.0,1.0)\\ \ln\;(\rho_{i+1}) & ∼\text{Normal}(\ln\;(\rho_{i}),\sigma_{i}\gamma \zeta ). \end{array}$$

The *HSMRF* model is an extension of the *GMRF* model with an additional local scale parameter for each interval, $\sigma_{i}$. This combination of local scale parameters $\sigma_{i}$ and global scale parameter *γ* allows the HSMRF model to produce generally more constant demographic history estimates but with occasional jumps. supplementary figure S18, Supplementary Material online shows the graphical model of the *HSMRF* model.

##### Skyfish

Our newly developed *Skyfish* model (derived from the french word poisson) is a variant of the *Bayesian multiple-change point* method (Opgen-Rhein et al. 2005). The *Skyfish* model assumes a Poisson prior distribution for the number of change-points and an autocorrelated normal prior distribution for each population size depending on the previous interval,


(14) $$\begin{array}{rl} H & =0.587405\\ \kappa & ∼\text{Poisson}(10.0)\\ \sigma & ∼\text{Exponential}(t_{MAX}/8)\\ \xi_{i} & ∼\text{Uniform}(0.0,t_{MAX})\\ \rho_{1} & ∼\text{Lognormal}(ln(t_{MAX}/4),sd=2\;*\;H)\\ \ln\;(\rho_{i+1}) & ∼\text{Normal}(\ln\;(\rho_{i}),\sigma (\xi_{i+1}-\xi_{i})). \end{array}$$

We assume a lognormal prior distribution on the first, i.e. most recent, population size with median $t_{MAX}/4$ and standard deviation 2*H where H=0.587405 so that 95% prior probability span two orders of magnitude (Höhna et al. 2017). The time $t_{MAX}$ is chosen as a good estimator for the maximum time of the most recent common ancestor for the samples, e.g. obtained from some preliminary analyses under a constant rate coalescent model (equation (6)) and adding some additional time to be conservative. We use an RJ-MCMC algorithm (Green 1995) to jointly infer the number of change points and demographic history. Specifically, we use the birth and death moves as described in May et al. (2016).

### Implementation of Bayesian Coalescent Skyline Plot Models in RevBayes

We implemented the Bayesian coalescent skyline plot models in the software RevBayes (Höhna et al. 2016). We provide one single interface to specify all Bayesian coalescent skyline plot models, dnCoalescentSkyline, computing the coalescent probability (equation (2)). This single distribution allows for consistent options between models while providing tremendous flexibility, for example, being applied to either isochronous or heterochronous data. The distribution takes the arguments (i) theta, (ii) times, (iii) events_per_interval, (iv) method, and (v) model. theta is the vector of per-interval population size values. This vector can either be sorted or unsorted, in which case the ordering is obtained by matching and sorting the times vector. The latter is useful for generic models where the number of change-points can change, for example, our *Skyfish* model. The vector of change-points times can be omitted if a coalescent event based change-point model is used (method=“events”). For the coalescent event based change-point models, however, the vector of events_per_interval is required. The interval change-point method can either be set to “events” in the case of coalescent event based models, or it can be set to “specified” in the case of coalescent event independent models. Finally, the argument model selects whether a *constant* or *linear* per-interval demographic function is assumed. Our implementation in RevBayes provides the full flexibility and range of applications supported by RevBayes, including posterior predictive simulations to assess model fit (Höhna et al. 2018; Duchêne et al. 2019; Cappello et al. 2020; Fonseca et al. 2022) and marginal-likelihood estimation to select between models (Höhna et al. 2021). Furthermore, RevBayes provides methods to either (i) jointly infer the genealogy and demographic history, (ii) infer the demographic history from a single given genealogy, e.g. the *maximum a posteriori* genealogy inferred from a constant population size analysis, or (iii) using a sample of genealogies from the posterior distribution of a previous analysis (sequential Bayesian inference).

### Empirical Example

We obtained a horse dataset from Vershinina et al. (2021) to explore and compare different Bayesian coalescent skyline plot models (e.g. autocorrelated vs. uncorrelated priors, coalescent event based intervals vs. independent intervals), inference approaches (sequential vs. joint inference) and data types (isochronous vs. heterochronous). The dataset comprises 173 mitochondrial genome sequences from different horse taxa. The samples are obtained from different points in time (heterochronous samples) with a subsample of size 36 being sampled at the present time (isochronous samples). For the analyses, we provided as data the multiple sequence alignment and the ages of the sequences; all being at the present for isochronous data and the empirical sampling times obtained from Vershinina et al. (2021) for the heterochronous data.

We performed Bayesian analyses using nine different coalescent skyline plot models (see above and Table 1). All models were run with the constant per-interval demographic function. Additionally, the *Skyfish* model was run with the linear per-interval demographic function. All coalescent event independent models which need specification of the number of intervals (*Skygrid*, *GMRF*, *HSMRF*) were run with 50 equally sized intervals. Furthermore, we tested both joint and sequential inference (Höhna and Hsiang 2024) by running the *Skyfish* analysis on (i) sequence data, (ii) the MAP genealogy (topology and coalescent times fixed) inferred from the constant population size analysis with sequence data, and (iii) a sample of either 10 or 100 genealogies inferred from the constant population size analysis with sequence data (sequential Bayesian inference). The sequential inference uses genealogy samples from the posterior distribution of a first analysis to estimate genealogies in a sequential fashion (i.e. two sequential analyses). The posterior samples of genealogies are treated as data in the second analysis and the likelihood is computed as an average reweighted by the prior probability on the genealogies of the original analysis (for specific details, see Höhna and Hsiang 2024). Note that the sequential Bayesian inference can only be performed for coalescent event independent intervals because different genealogy samples have different coalescent times and therefore different demographic histories despite the same population size parameters. For our newly developed *Skyfish* model, we additionally tested three different prior settings: an autocorrelated version, and two uncorrelated versions with one of them having an empirically informed prior.

We performed all analyses on both datasets, the complete heterochronous dataset and the isochronous subsample. For each MCMC analysis, we set the number of replicate runs to two and ran the MCMC for 100,000 iterations. We sampled every 10th iteration and applied a burn-in of 10% in the evaluation, leading to 18,000 samples per analysis in total. We checked for convergence using custom R scripts by comparing the difference in cumulated distribution function of the demographic history (Fabreti and Höhna 2022). Additionally, we estimated marginal likelihoods for each model for both datasets using the stepping stone sampler (Xie et al. 2011; Baele et al. 2012; Höhna et al. 2021). We ran 127 power posterior analyses using default spacing of powers (Höhna et al. 2021) and 5,000 iterations each.

### Simulation Study

In our empirical analyses, we obtained two types of qualitatively different results: a rather constant demographic history and a more complex demographic history with a decline towards the past. We explored the different models in a simulation study where we knew the true demographic history. This simulation study investigates if this difference is either due to lack of power to detect the population size change or due to over-sensitivity (i.e. false positive results). We simulated 36 isochronous sequences following the median population size trajectory from the *BSP* analysis and the *Constant* analysis (isochronous samples and with the constant per-interval demographic function, see Fig. 1). These trajectories were chosen for simulation as representatives of the two types of results we obtained in the empirical analyses. Each simulation setting was used for 10 simulations. For the resulting 20 simulated datasets, we then performed analyses under the *BSP* and *Skyfish* models to see which of the models would best capture the simulated population size trajectory.

### Validation Study

In our simulation study, we found that the *BSP* model inferred population size variation even if the true population size was constant. To rule out implementation problems, we performed four *BSP* analyses with BEAST and RevBayes on the same simulated datasets with the same priors and MCMC settings. Each *BSP* analysis was run for 25,000,000 iterations, sampling every ${1,000}^{th}$ generation, leading to 25,000 samples from the posterior distribution. For evaluation of the resulting trajectories, a burn-in of 25% was chosen.

## Supplementary Material

msae073_Supplementary_Data

## Data Availability

Scripts and data for running the described analyses are available at: https://github.com/rbillenstein/comp_skyline_models. A tutorial can be found at: https://revbayes.github.io/tutorials/coalescent/. Mitochondrial sequences were taken from Vershinina et al. (2021).
