# Supplementary material for: Comparison of Bayesian Coalescent Skyline Plot Models for Inferring Demographic Histories
Source: Mol Biol Evol. 2024 Apr 17;41(5):msae073. doi: 10.1093/molbev/msae073 (PMC11068272; doi:10.1093/molbev/msae073)
Supplement: msae073_Supplementary_Data [file msae073_supplementary_data.pdf]

Comparison of Bayesian Coalescent Skyline Plot Models for Inferring Demographic Histories

Supporting Information

Ronja J. Billenstein[1,2] and Sebastian Höhna[1,2,*]

[1] *GeoBio-Center, Ludwig-Maximilians-Universität München, Richard-Wagner Straße 10, 80333 Munich, Germany*
[2] *Department of Earth and Environmental Sciences, Paleontology & Geobiology, Ludwig-Maximilians-Universität München, Richard-Wagner Straße 10, 80333 Munich, Germany*
[*] *To whom correspondence should be addressed: Sebastian.Hoehna@gmail.com*

Contents

S1 Marginal likelihoods of the models **3**

S2 Sequence based analyses **4**
S2.1 Estimated demographic histories . . . 5
S2.2 Convergence assessment . . . 7

S3 Simulation Study **9**
S3.1 Simulations using the results of the *BSP* analysis to simulate . . . 9
S3.2 Simulations using the results of the *Constant* analysis to simulate . . . 10

S4 Comparison of joint and sequential inference **11**
S4.1 Estimated demographic histories . . . 11
S4.2 Convergence assessment . . . 12

S5 Comparison of constant and linearly changing per-interval population size **13**
S5.1 Estimated demographic histories . . . 13
S5.2 Convergence assessment . . . 14

S6 Comparison of autocorrelated and uncorrelated priors for the *Skyfish* model **15**
S6.1 Estimated demographic histories . . . 15
S6.2 Convergence assessment . . . 16

S7 Validation by comparison between `RevBayes` and `BEAST` **17**

S8 Number of change-points inferred from the *Skyfish* model **19**

S9 Lineages through time **20**

S10 Graphical models **21**

S1 Marginal likelihoods of the models

We performed marginal likelihood estimation using stepping-stone sampling [9] using the parallel power posterior sampler in `RevBayes` [5]. Marginal likelihoods were estimated using the same models and data settings as described in the main manuscript. The power posterior MCMC analyses were run for 128 stones with 10,000 MCMC iterations as burnin and then 2,000 iterations for each consecutive stone.

Table S1: Overview of log-transformed marginal likelihoods estimated with a stepping-stone algorithm. Highest support is highlighted with bold font.

model	isochronous data	heterochronous data
BSP	-26981.50	-41652.75
Constant	-26980.93	-41667.40
EBSP	-26987.58	-41660.42
GMRF	-26980.21	-41672.13
HSMRF	-26981.56	-41644.25
Skyfish	**-26979.01**	-41654.25
Skygrid	-26984.14	**-41641.27**
Skyline	-26984.57	-41722.49
Skyride	-26983.48	-41643.22

S2 Sequence based analyses

We performed demographic analyses of horse data [8] with nine different models: a *Constant* model, a *Skyline* model, a Bayesian Skyline Plot (*BSP*) model [1], an Extended BSP (*EBSP*) model [4], a *Skyride* model [6], a *Skygrid* model [3], a Gaussian Markov Random Field (*GMRF*) model [2], a Horseshoe Markov Random Field (*HSMRF*) model [2], and our new *Skyfish* model (similar to [7]). Here, we show the resulting population size trajectories on a timeline from 0 to $1,200,000$ years ago and the convergence assessment comparing two replicates of each analysis. The convergence assessment spans the time of the original analyses ($0 - 500,000$ years ago for the isochronous samples, $0 - 1,200,000$ years ago for the heterochronous samples).

S2.1 Estimated demographic histories

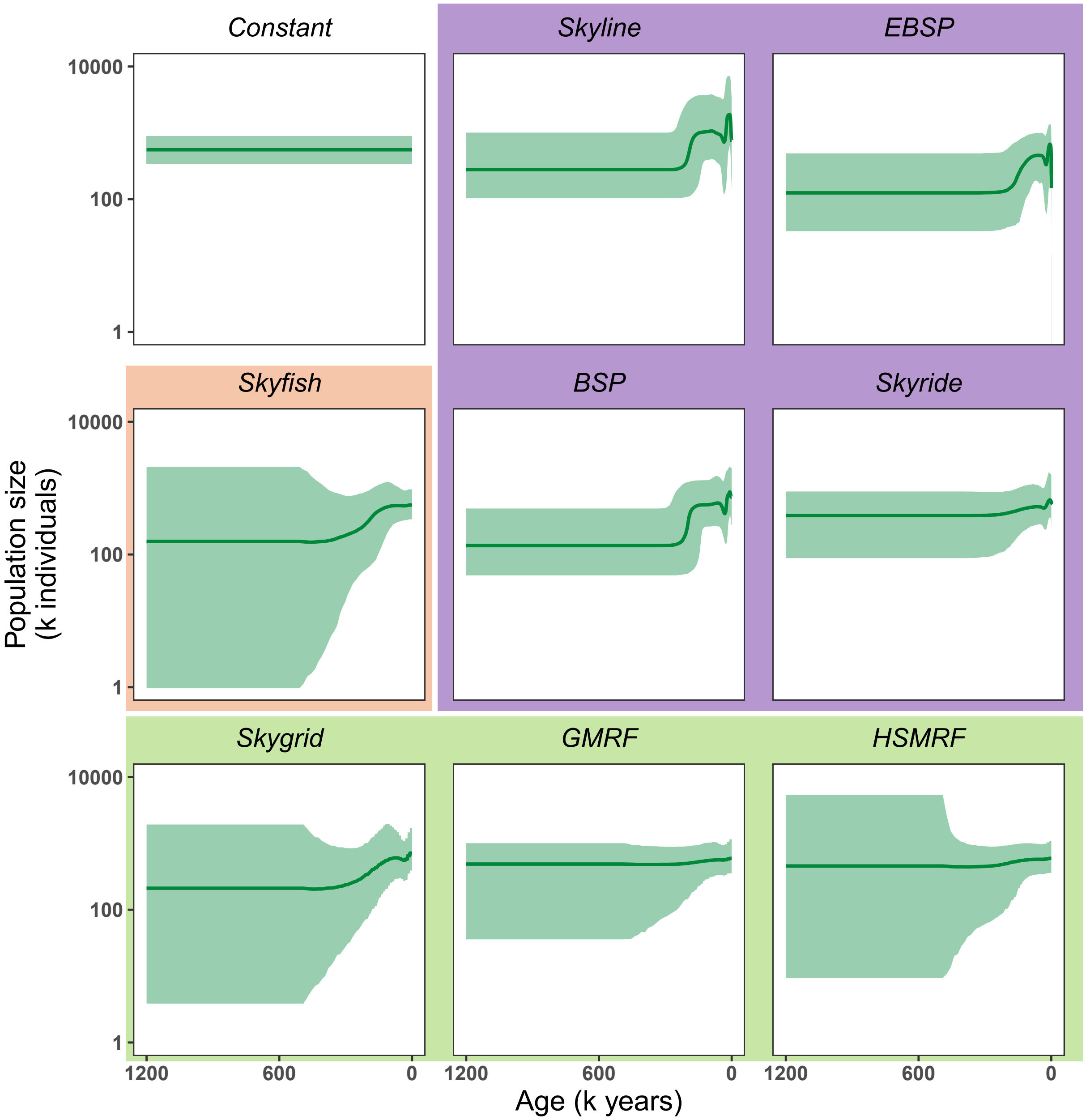

Figure S1: Population size trajectories estimated with nine different models from isochronous sequence data on a timeline from 0 to 1, 200, 000 years ago. Genealogies and population size were jointly estimated. All analyses considered possible variation in population size between 0 and 500, 000 years ago. The bold line represents the median of the posterior distribution of the population size and the shaded area shows the 95% credible intervals. The models highlighted in violet (*BSP*, *EBSP*, *Skyline*, *Skyride*) are coalescent event based models, i.e., a change in population size can only occur at the time of a coalescent event. In models highlighted in green, population size changes can happen at specified times, independent from coalescent events. Here, all intervals for these models are equally-sized. In the orange *Skyfish* model, the number of intervals as well as their duration are estimated. All models except for the *Constant* model, the *EBSP* model, and the *Skyline* model have correlated intervals. MCMCs were run for 100, 000 iterations, sampling every tenth iteration, with a burn-in of 10% and two replicates, yielding 18, 000 samples in total. For plotting, the resulting trajectories were evaluated at 500 exponentially-spaced grid points between 0 and 1, 200, 000 years ago.

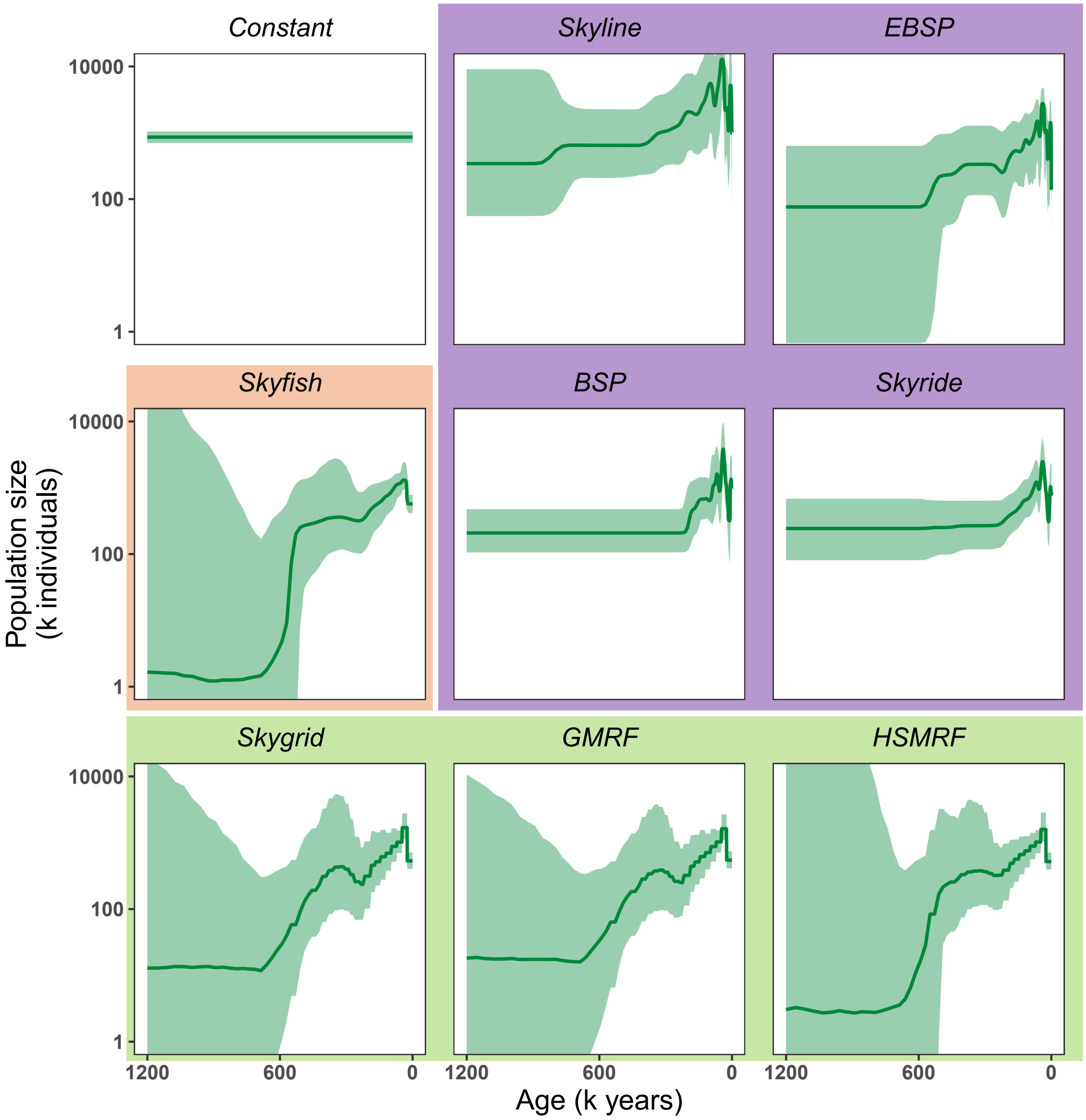

Figure S2: Population size trajectories estimated with nine different models from heterochronous sequence data on a timeline from 0 to 1,200,000 years ago. Genealogies and population size were jointly estimated. All analyses considered possible variation in population size between 0 and 1,200,000 years ago. The bold line represents the median of the posterior distribution of the population size and the shaded area shows the 95% credible intervals. The models highlighted in violet (*BSP*, *EBSP*, *Skyline*, *Skyride*) are coalescent event based models, i.e., a change in population size can only occur at the time of a coalescent event. In models highlighted in green, population size changes can happen at specified times, independent from coalescent events. Here, all intervals for these models are equally-sized. In the orange *Skyfish* model, the number of intervals as well as their duration are estimated. All models except for the *Constant* model, the *EBSP* model, and the *Skyline* model have correlated intervals. MCMCs were run for 100,000 iterations, sampling every tenth iteration, with a burn-in of 10% and two replicates, yielding 18,000 samples in total. For plotting, the resulting trajectories were evaluated at 500 exponentially-spaced grid points between 0 and 1,200,000 years ago.

S2.2 Convergence assessment

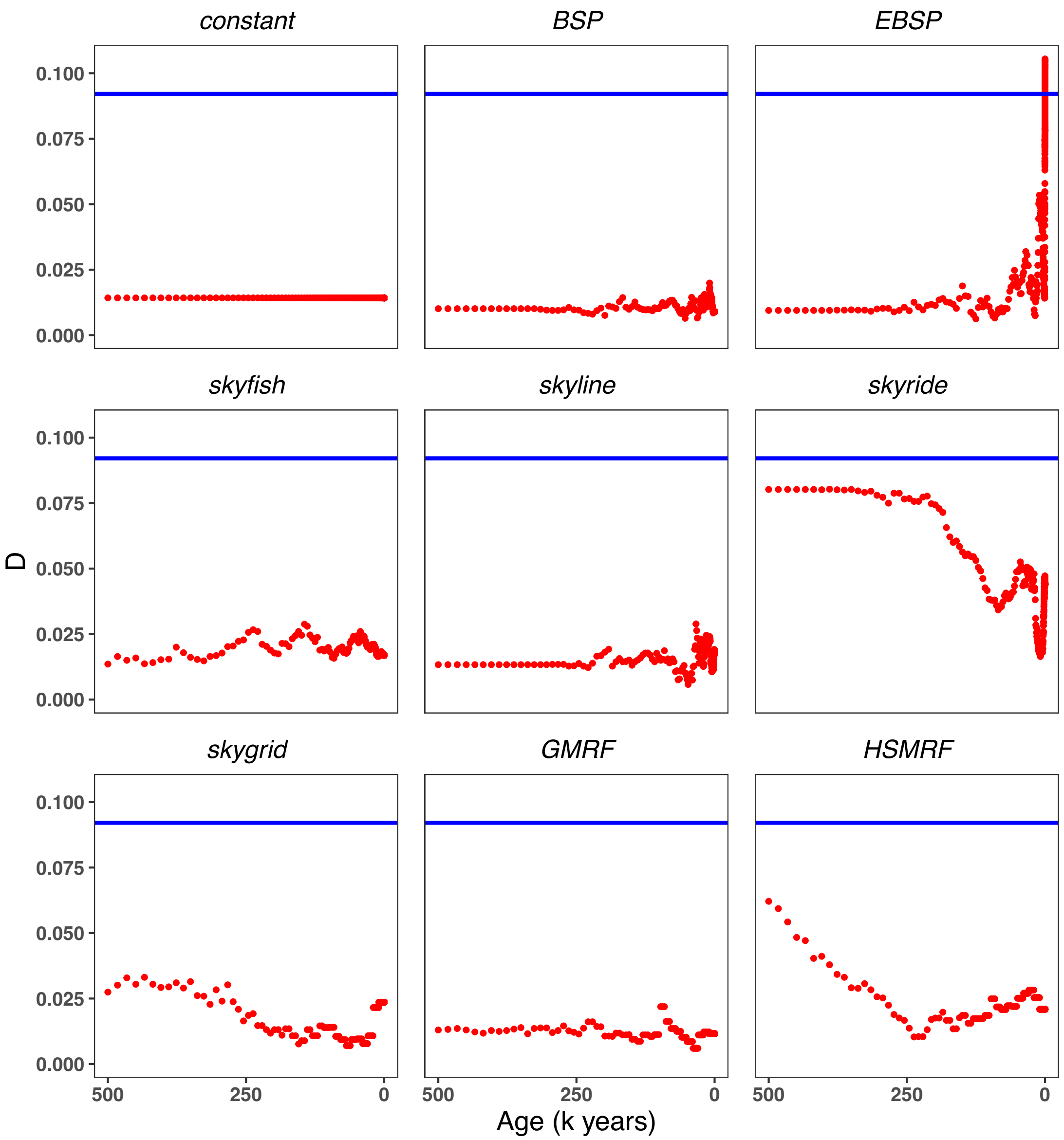

Figure S3: Convergence assessment of analyses with isochronous sequence data. Kolmogorov-Smirnov test statistic (D) was calculated for the posterior distributions of two independent MCMC runs at 500 exponentially spaced grid points. The solid blue line depicts the threshold of 0.0921.

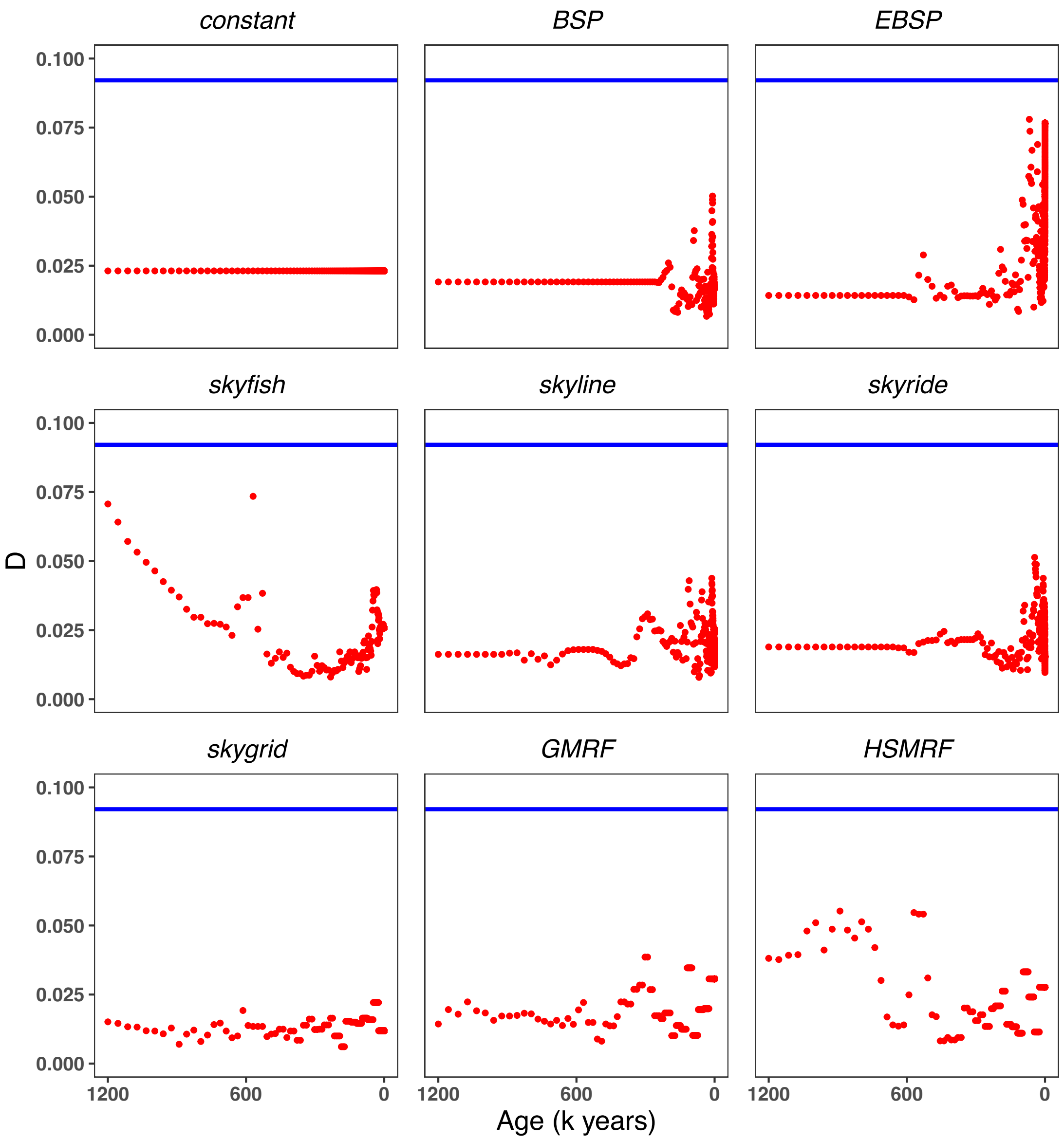

Figure S4: Convergence assessment of analyses with heterochronous sequence data. Kolmogorov-Smirnov test statistic (D) was calculated for the posterior distributions of two independent MCMC runs at 500 exponentially spaced grid points. The solid blue line depicts the threshold of 0.0921.

S3 Simulation Study

S3.1 Simulations using the results of the *BSP* analysis to simulate

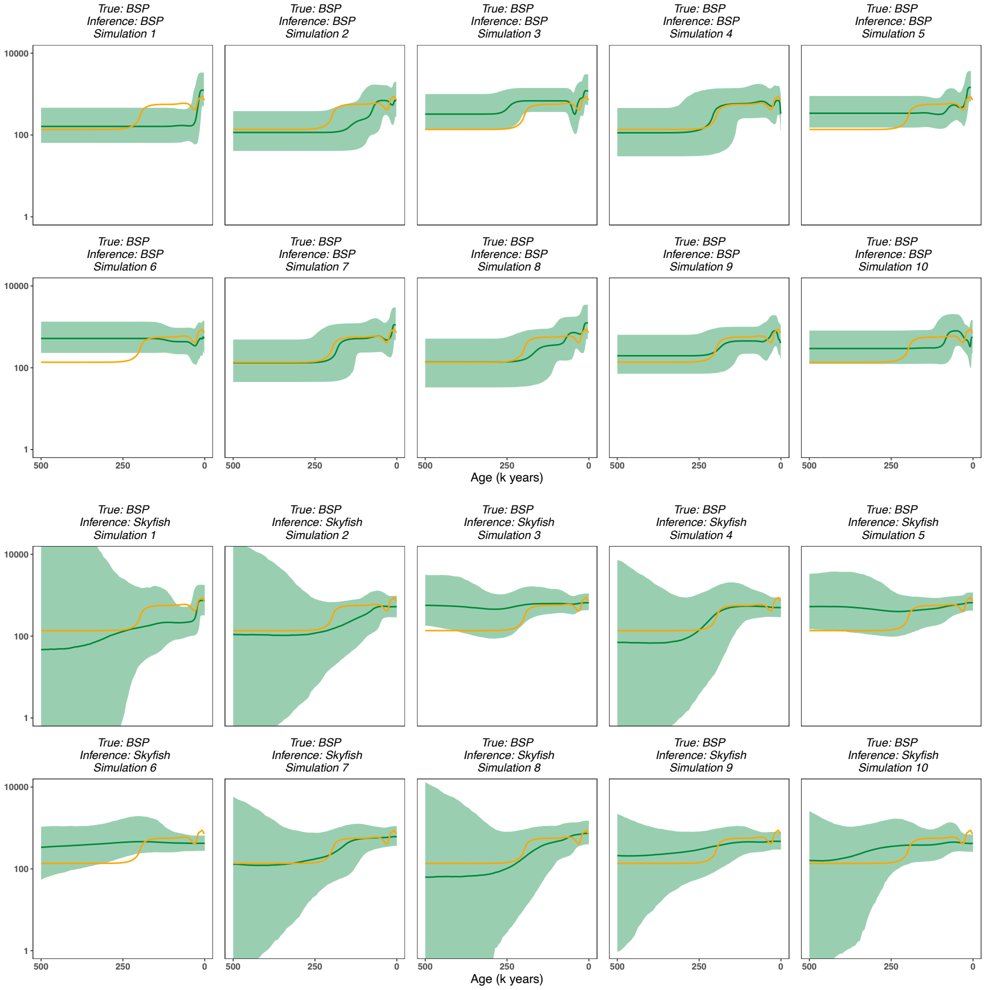

Figure S5: Simulation study results, simulated under the population size trajectory from the *BSP* analysis. 10 simulations of sequence data with 36 tips were performed under the resulting population size trajectory from the *BSP* analysis with isochronous data. Analyses of each of the simulations were performed with the *BSP* model (top two rows) and the *Skyfish* model (bottom two rows). The bold green line represents the median of the posterior distribution of the population size and the shaded area shows the 95% credible intervals. The true trajectory used for simulations is depicted in orange.

S3.2 Simulations using the results of the *Constant* analysis to simulate

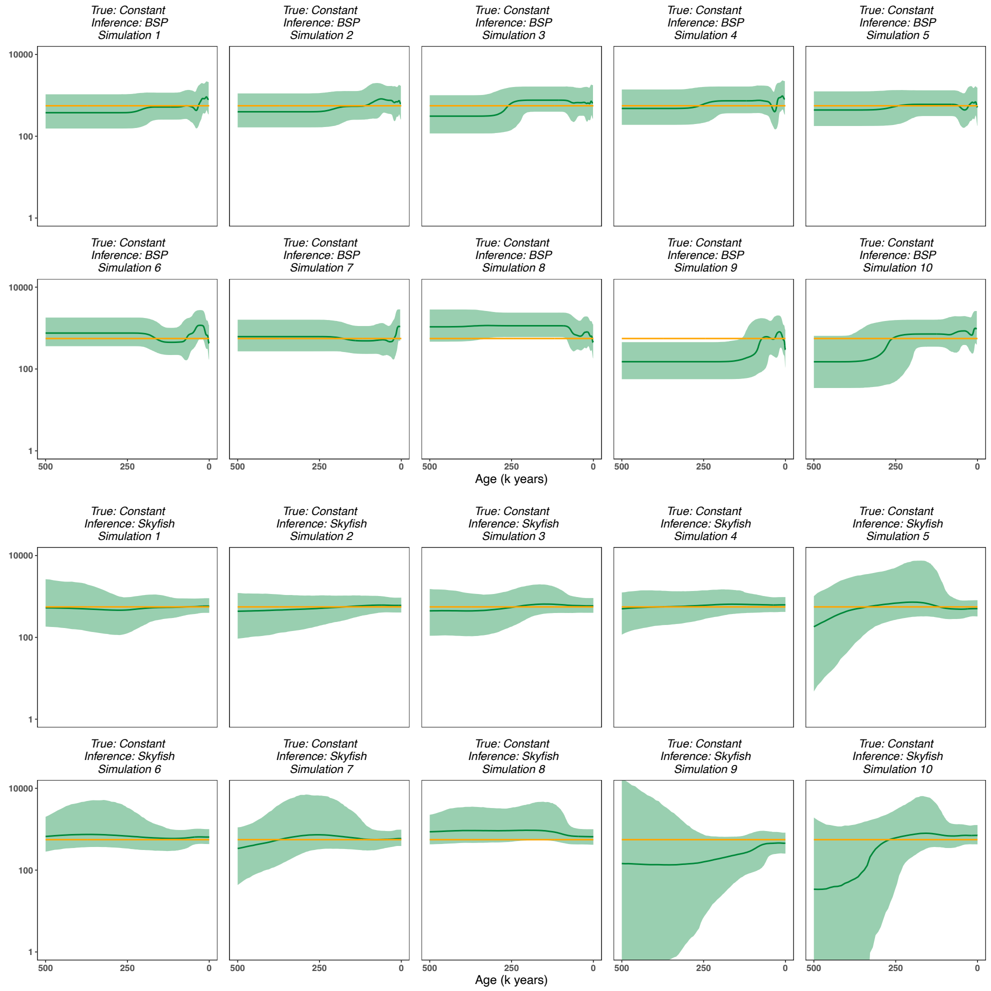

Figure S6: Simulation study results, simulated under the population size trajectory from the *Constant* analysis. 10 simulations of sequence data with 36 tips were performed under the resulting population size trajectory from the *Constant* analysis with isochronous data. Analyses of each of the simulations were performed with the *BSP* model (top two rows) and the *Skyfish* model (bottom two rows). The bold green line represents the median of the posterior distribution of the population size and the shaded area shows the 95% credible intervals. The true trajectory used for simulations is depicted in orange.

S4 Comparison of joint and sequential inference

S4.1 Estimated demographic histories

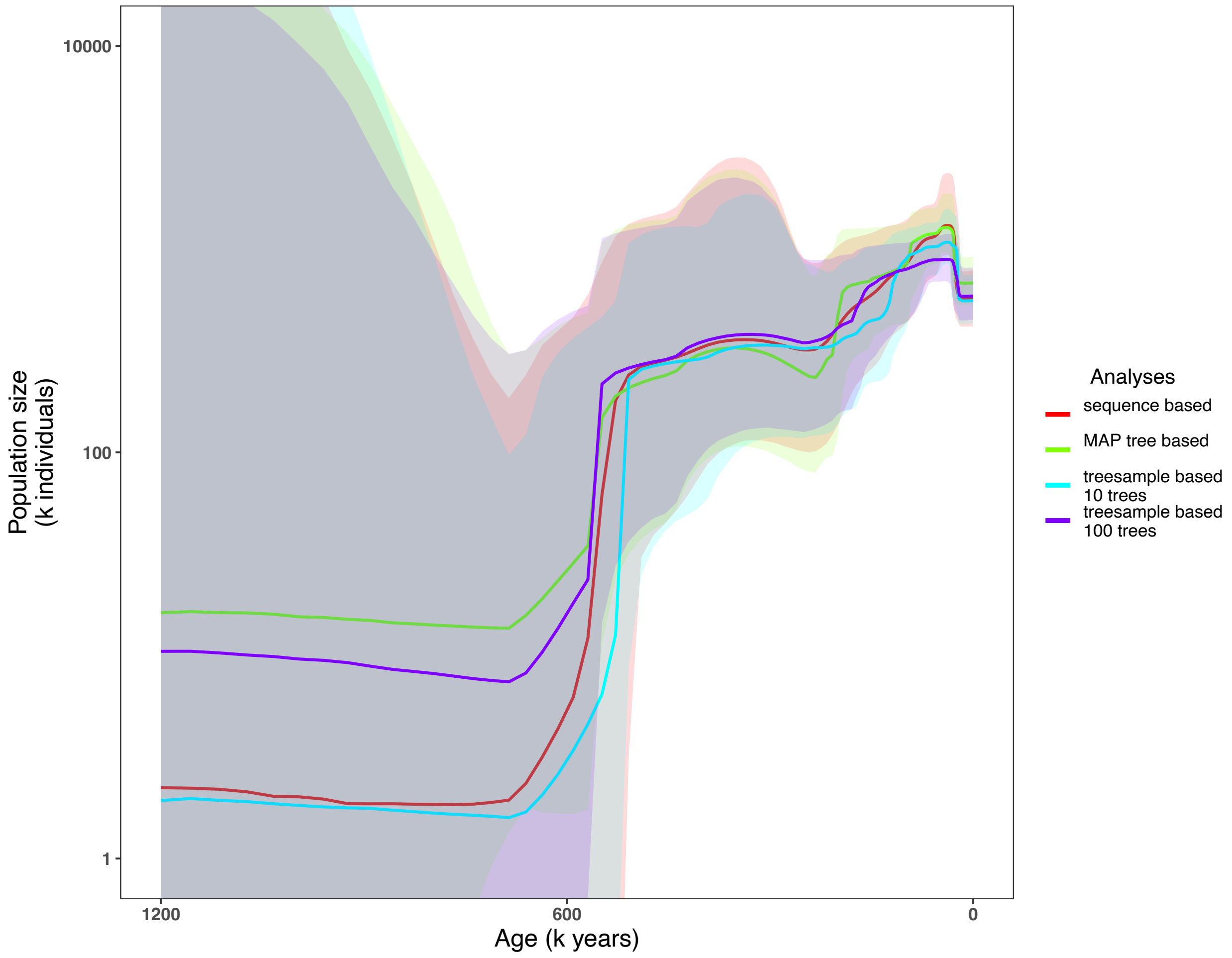

Figure S7: Comparing joint and sequential inference of population size trajectories with the *Skyfish* model from heterochronous data. The *Skyfish* analysis was run between 0 and $1,200,000$ years ago. Red: Sequence based analysis from the original data. Green: MAP tree based analysis using the MAP tree from the *Constant* analysis with sequence data. Light blue: Analysis based on 10 trees from the posterior distribution of the *Constant* analysis with sequence data. Violet: Analysis based on 100 trees from the posterior distribution of the *Constant* analysis with sequence data. The bold lines represents the medians of the posterior distributions of the population size and the shaded areas show the 95% credible intervals.

S4.2 Convergence assessment

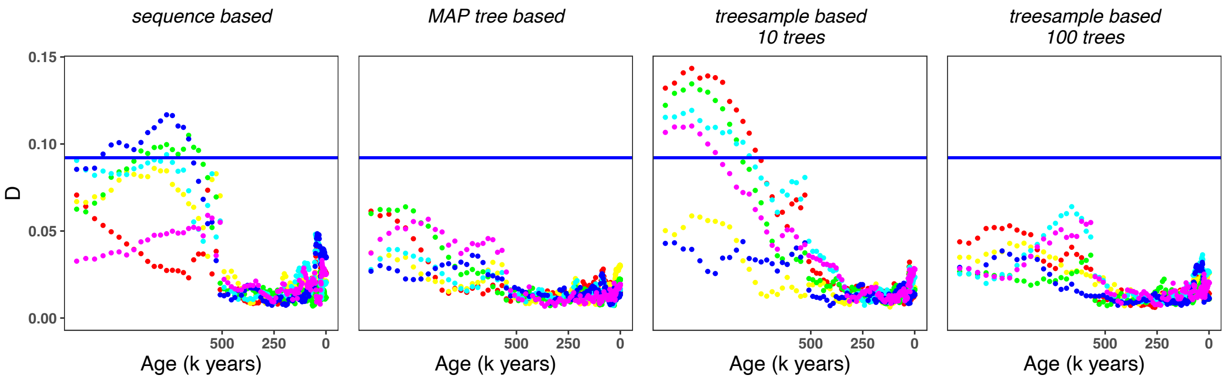

Figure S8: Convergence of *Skyfish* analyses, comparing joint and sequential inference of population size trajectories from heterochronous data. From left to right: sequence based analysis from the original data; MAP tree based analysis using the MAP tree from the *Constant* analysis with sequence data; analysis based on 10 trees from the posterior distribution of the *Constant* analysis with sequence data; analysis based on 100 trees from the posterior distribution of the *Constant* analysis with sequence data. Kolmogorov-Smirnov test statistic (D) was calculated for the posterior distributions of four independent MCMC runs at 500 exponentially spaced grid points. The solid blue line depicts the threshold of 0.0921.

S5 Comparison of constant and linearly changing per-interval population size

S5.1 Estimated demographic histories

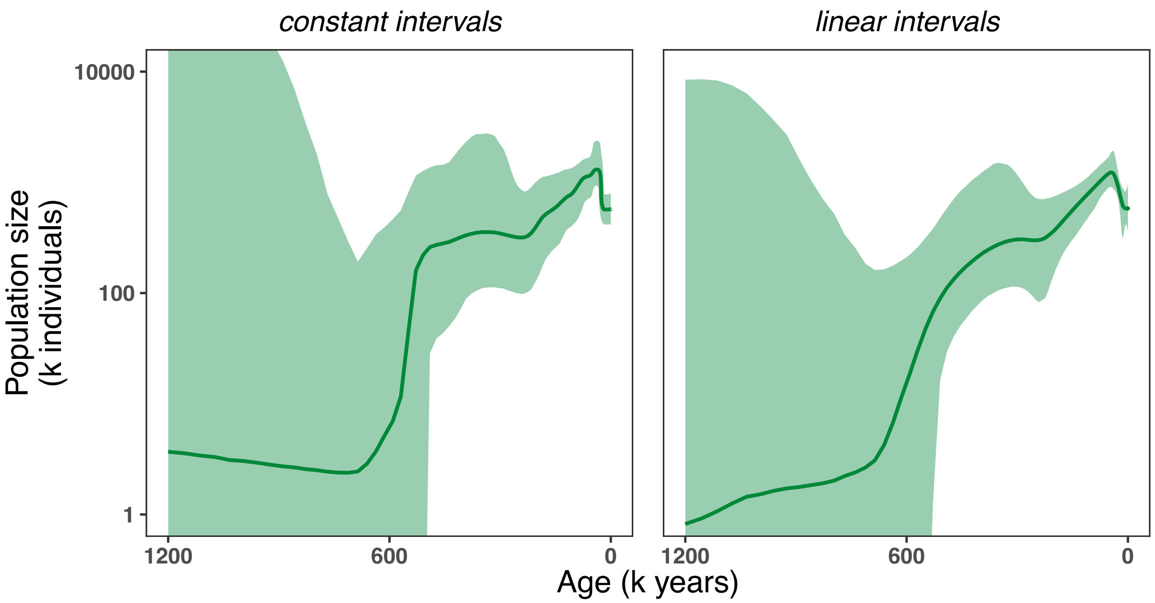

Figure S9: Comparing constant and linearly changing population size within intervals with the *Skyfish* model from heterochronous data. The bold line represents the median of the posterior distribution of the population size and the shaded area shows the 95% credible intervals.

S5.2 Convergence assessment

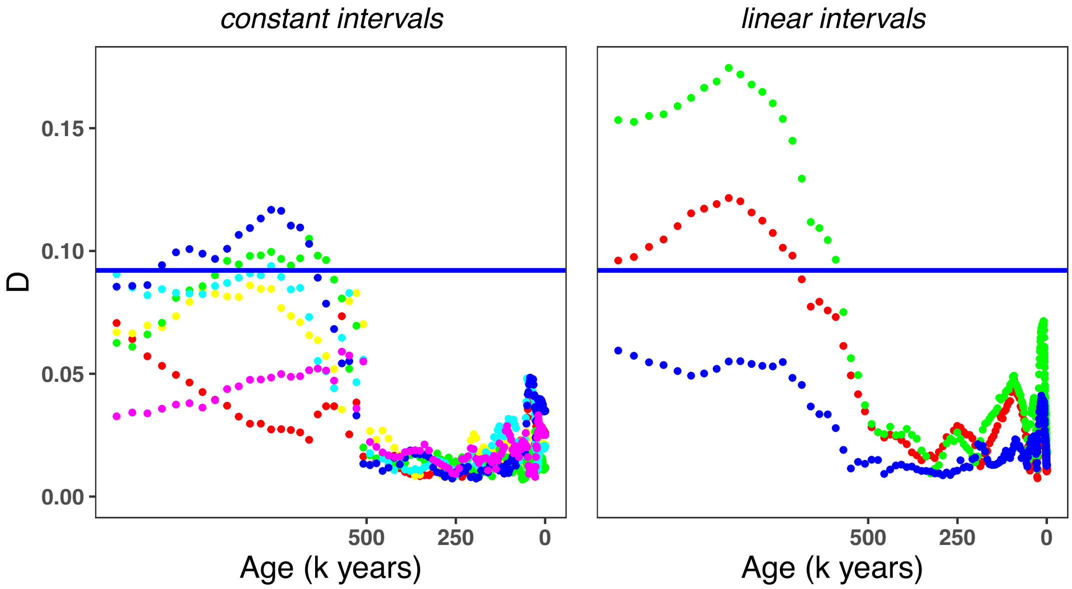

Figure S10: Convergence of *Skyfish* analyses, comparing constant and linearly changing population size within intervals. Kolmogorov-Smirnov test statistic (D) was calculated for the posterior distributions of four independent MCMC runs at 500 exponentially spaced grid points. For linearly changing population size, one replicate did not converge and was excluded. The solid blue line depicts the threshold of 0.0921

S6 Comparison of autocorrelated and uncorrelated priors for the *Skyfish* model

S6.1 Estimated demographic histories

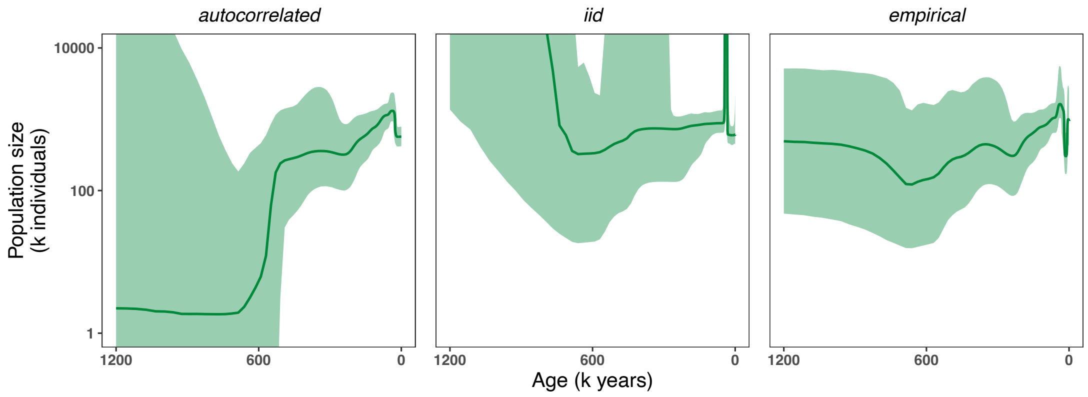

Figure S11: Comparing different *Skyfish* models for sequence based analyses from heterochronous data. From left to right: *Skyfish* analysis with autocorrelated population size values; *Skyfish* analysis with iid intervals; *Skyfish* analysis with iid intervals and an empirically informed prior. The bold line represents the median of the posterior distribution of the population size and the shaded area shows the 95% credible intervals.

S6.2 Convergence assessment

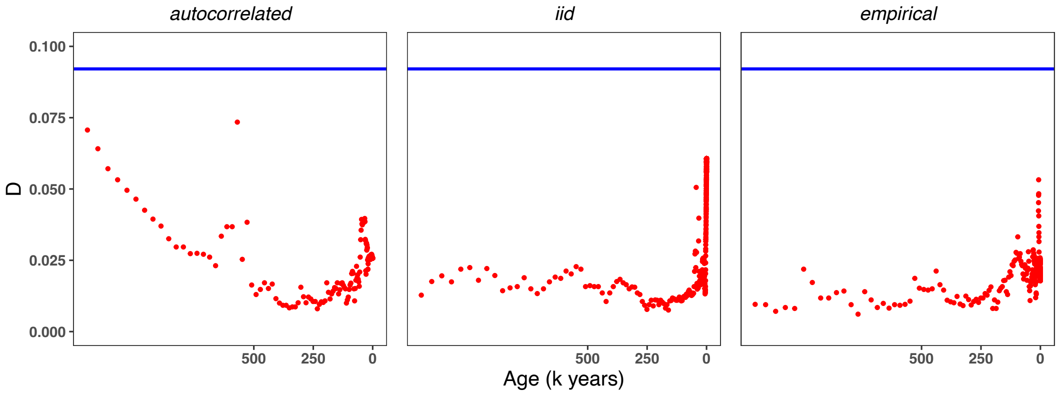

Figure S12: Convergence of different *Skyfish* models for sequence based analyses from heterochronous data. From left to right: *Skyfish* analysis with autocorrelated population size values; *Skyfish* analysis with iid intervals; *Skyfish* analysis with iid intervals and an empirically informed prior. Kolmogorov-Smirnov test statistic (D) was calculated for the posterior distributions of two independent MCMC runs at 500 exponentially spaced grid points. The solid blue line depicts the threshold of 0.0921

S7 Validation by comparison between RevBayes and BEAST

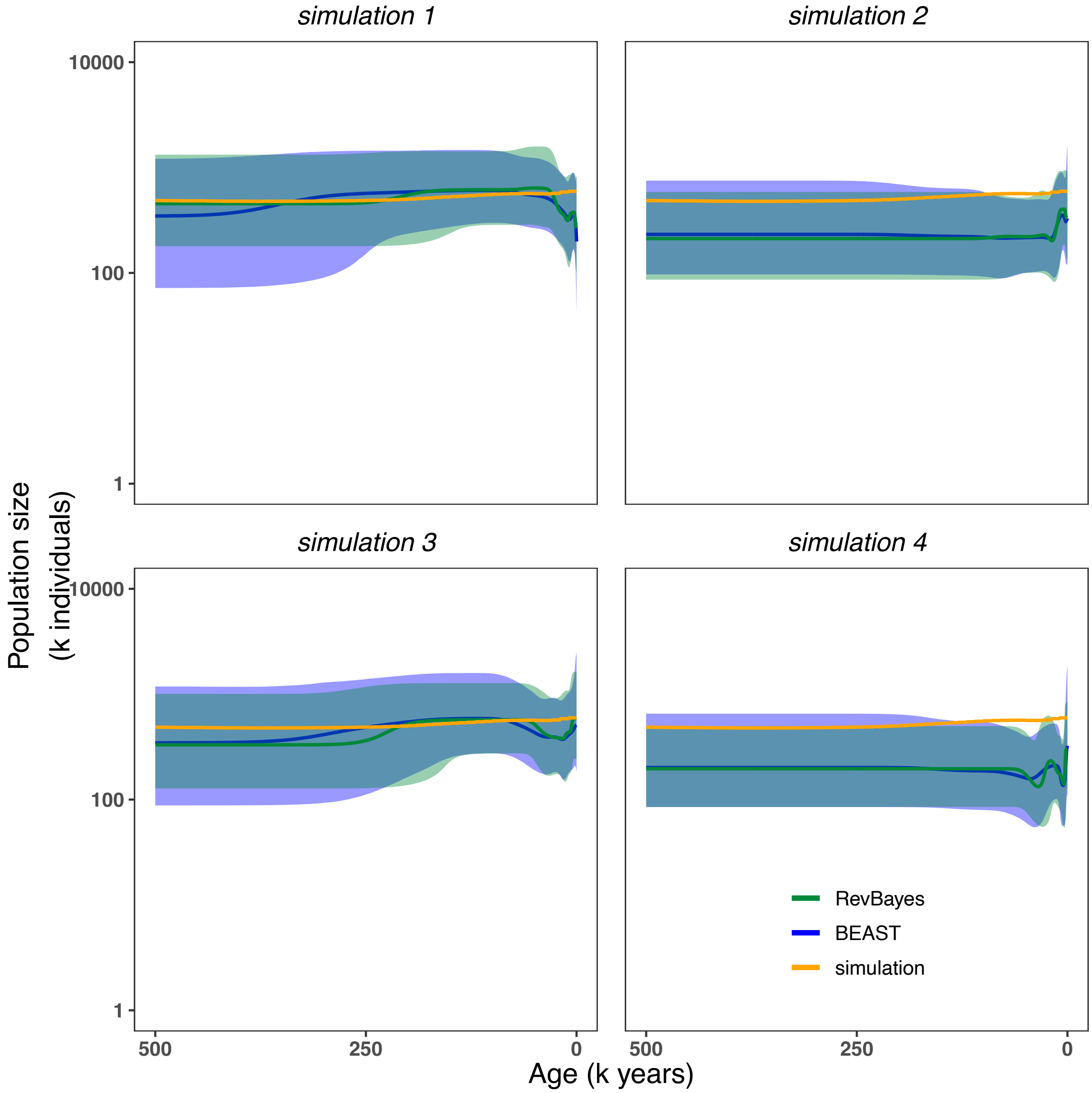

Figure S13: Comparing *BSP* analyses on simulated data from 4 simulations. Blue: `BEAST1`. Green: `RevBayes`. Orange: True population size trajectory used for simulations. The bold line represents the median of the posterior distribution of the population size and the shaded area shows the 95% credible intervals.

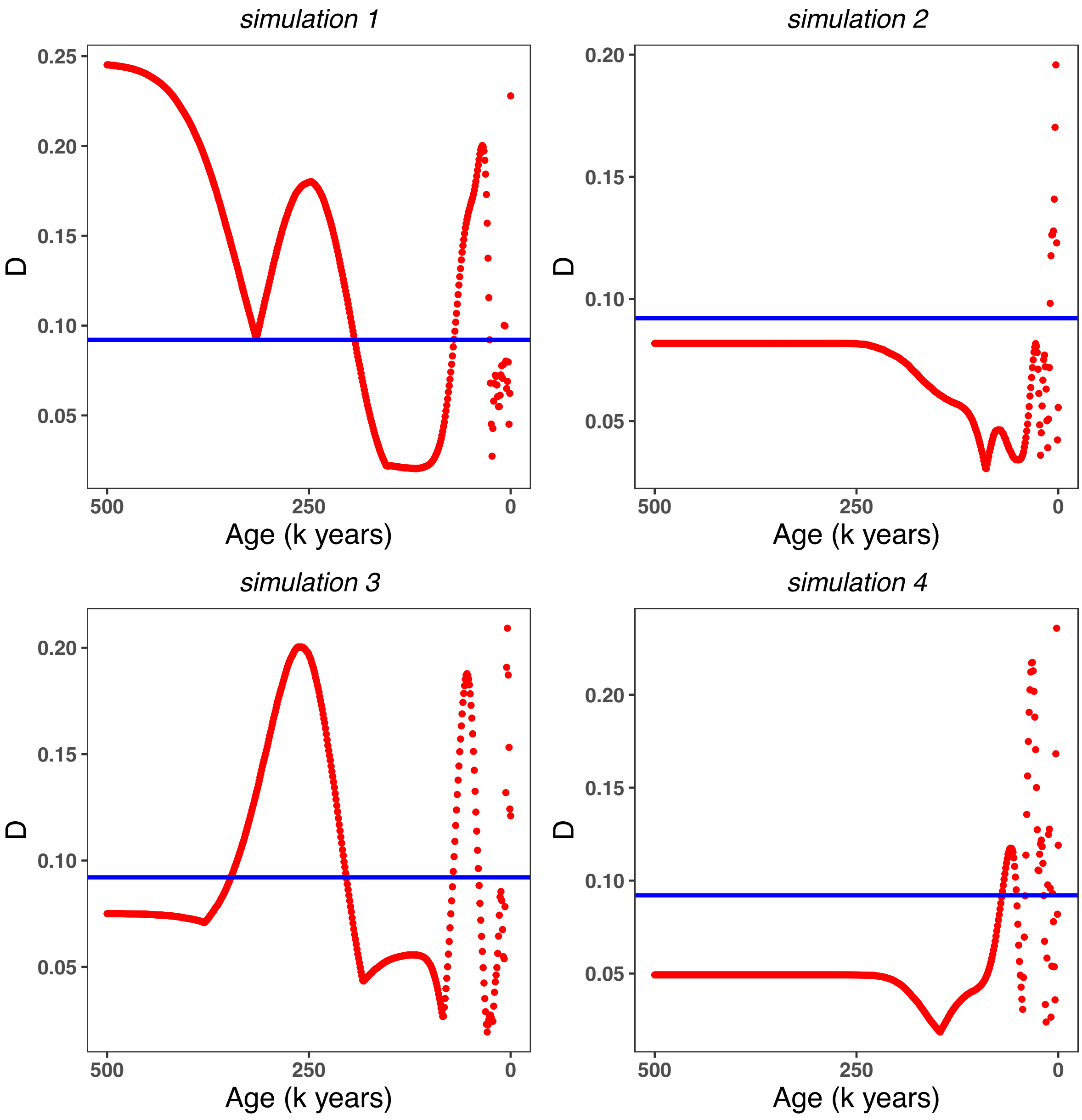

Figure S14: Kolmogorov-Smirnov test statistic comparing the posterior distributions of *BSP* analyses with `BEAST1` and `RevBayes` at 500 equally spaced grid points. The solid blue line depicts the threshold of 0.0921.

S8 Number of change-points inferred from the *Skyfish* model

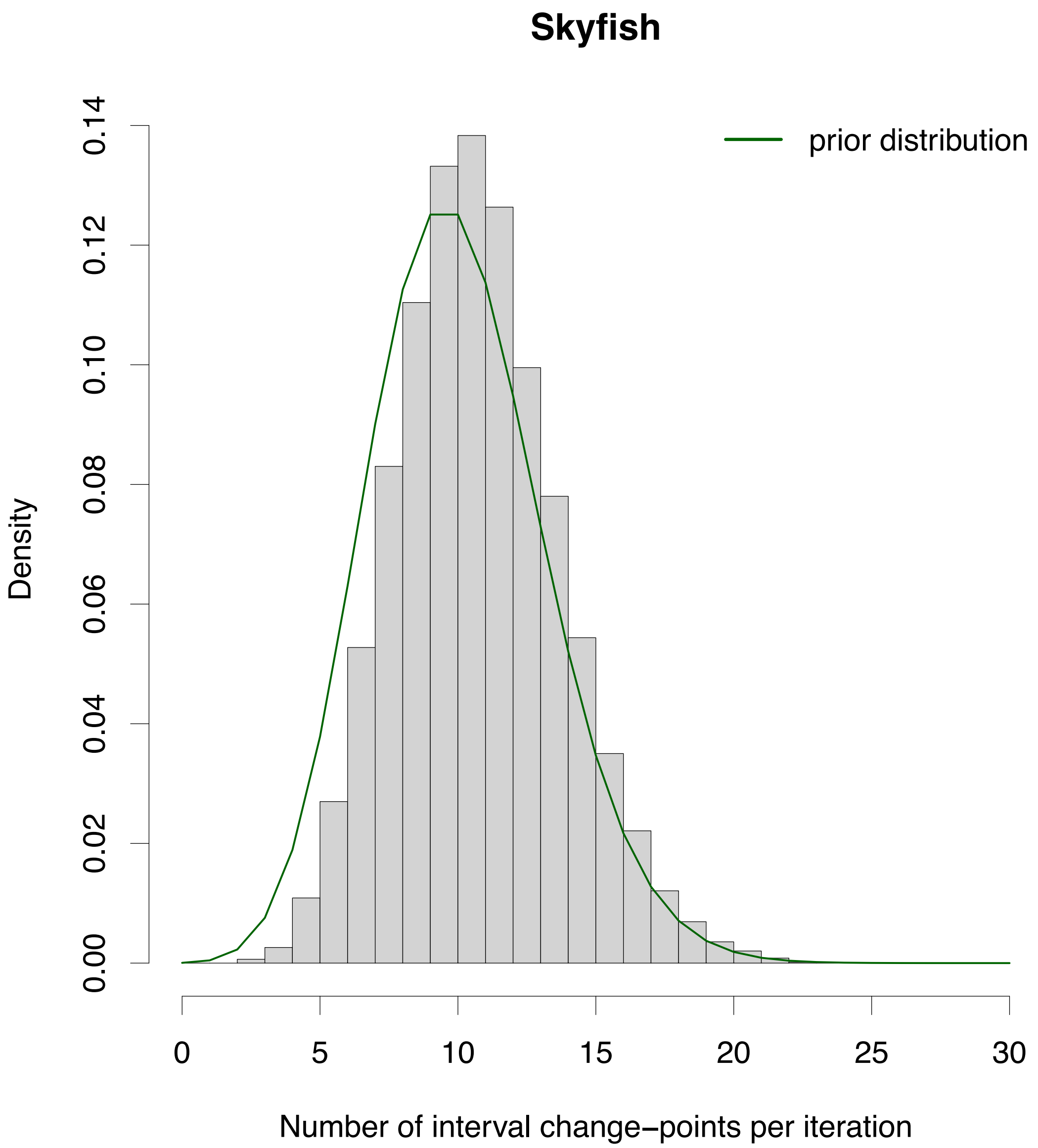

Figure S15: Histogram of the number of change-points encountered in an analysis with the *Skyfish* model. The analysis used heterochronous sequence data as input and a constant population size within intervals. It was run with four replicates for 100,000 iterations each, sampling every tenth iteration. For the histogram, the first 10% of samples were discarded, yielding 36,000 samples. The solid green line depicts the prior distribution, a Poisson distribution with a mean of ten.

S9 Lineages through time

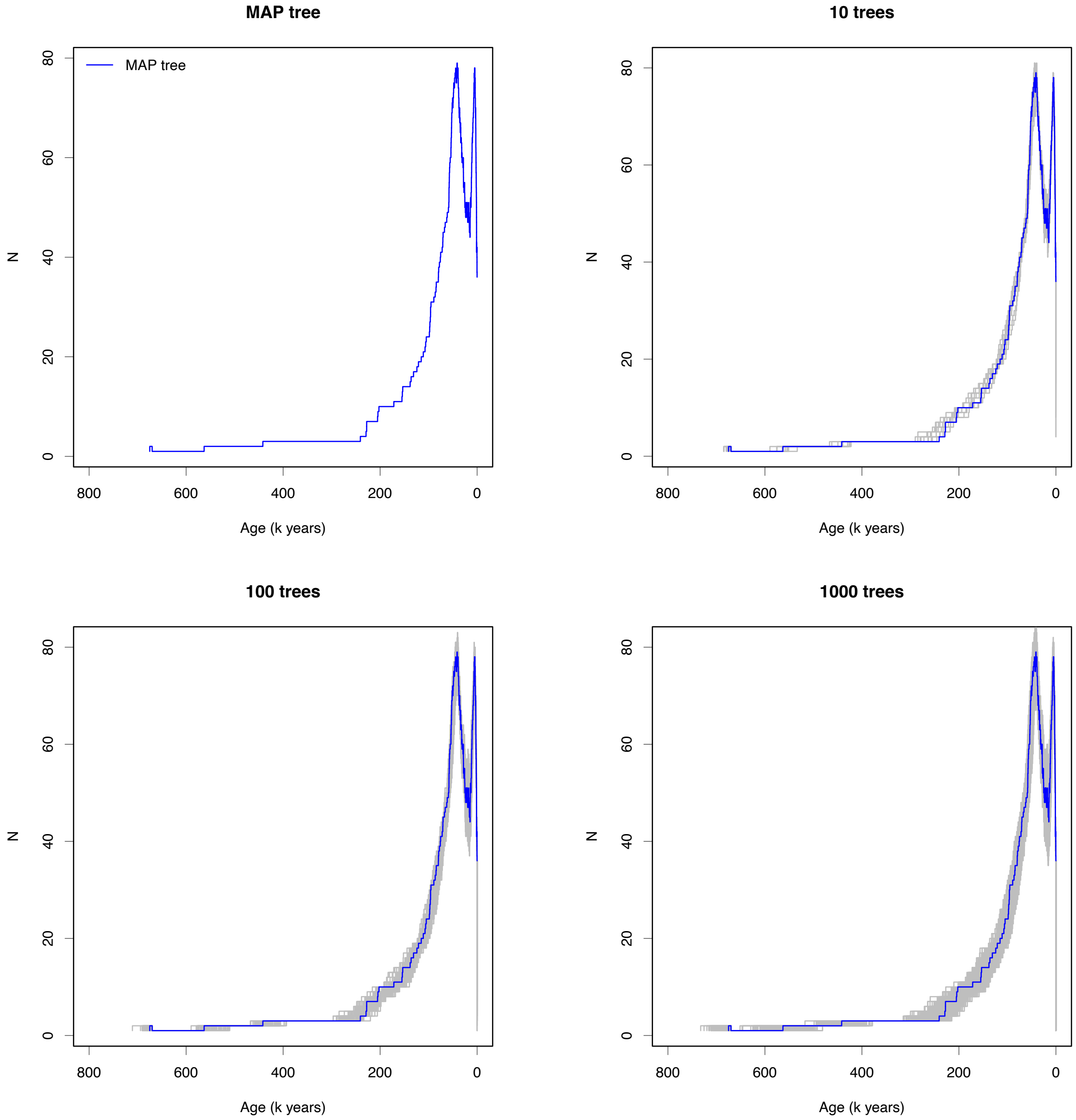

Figure S16: Lineages through time (LTT) plots of trees from an analysis of heterochronous sequence data with the *Constant* model. The analysis was run with four replicates for 100,000 iterations, sampling every tenth iteration. The MAP tree was calculated from the posterior distribution of trees with a burn-in of 10%, i.e., from 36,000 trees. The three additional plots show LTT curves from a growing number of subsamples from this distribution of trees.

S10 Graphical models

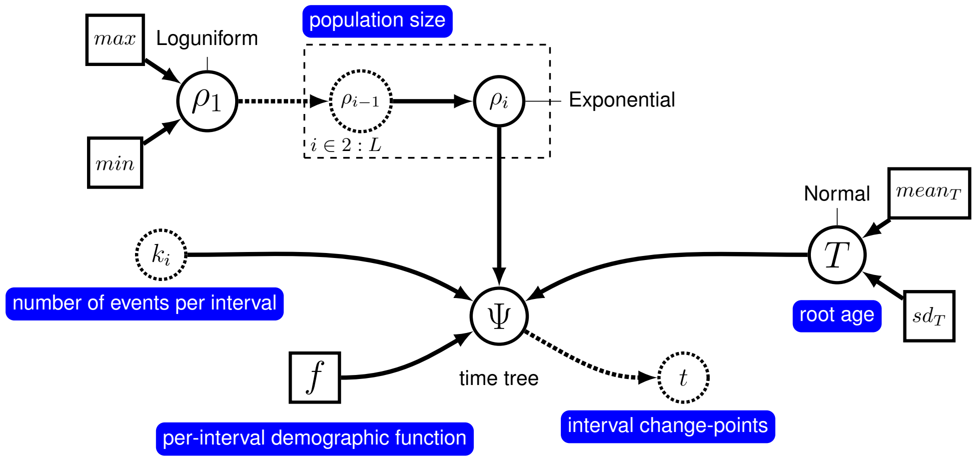

Figure S17: Graphical model of the *BSP* model used in this study. The population size values ρ are autocorrelated through an exponential distribution on the previous population size. The population size of the first interval follows a loguniform distribution. The number of events per interval k_i needs to be provided as input. The per-interval demographic function f (i.e. constant or linearly changing) needs to be provided as well. The root age T is normally distributed with mean $mean_T$ and standard deviation sd_T. Please note that interval change-points t coincide with coalescent events and are thus determined from the time tree based on k_i.

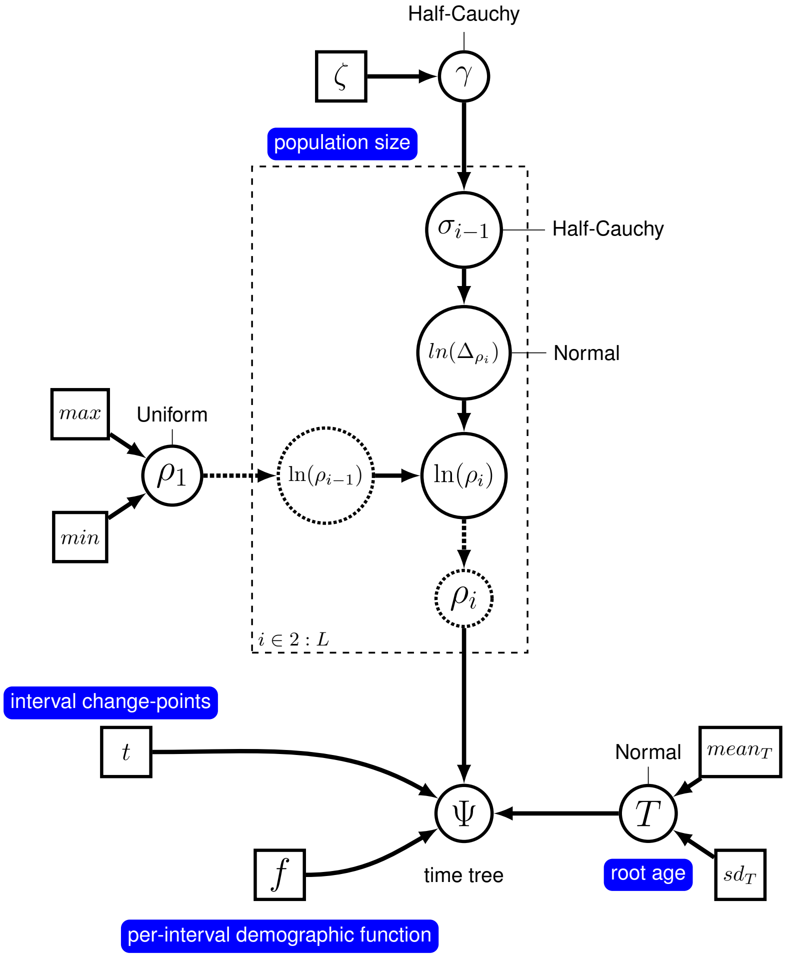

Figure S18: Graphical model of the *HSMRF* model used in this study. The population size values ρ are autocorrelated through log-transformed rates. The log differences $ln(\Delta)$ have a standard deviation of $\sigma_i \gamma \zeta$ with σ_i being the local scale for each interval, γ being the global scale, and ζ being the smoothing parameter. The standard deviation of the Normal distribution of the log differences thus has a hyperprior distribution. The population size of the first interval is uniformly distributed. The interval change-points t are fixed and provided as input. The per-interval demographic function f (i.e. constant or linearly changing) needs to be provided as well. The root age T is normally distributed with mean $mean_T$ and standard deviation sd_T.

References

[1] Drummond, A. J., Rambaut, A., Shapiro, B., and Pybus, O. G. 2005. Bayesian Coalescent Inference of Past Population Dynamics from Molecular Sequences. *Molecular Biology and Evolution*, 22(5): 1185–1192.

[2] Faulkner, J. R., Magee, A. F., Shapiro, B., and Minin, V. N. 2020. Horseshoe-based Bayesian nonparametric estimation of effective population size trajectories. *Biometrics*, 76(3): 677–690.

[3] Gill, M. S., Lemey, P., Faria, N. R., Rambaut, A., Shapiro, B., and Suchard, M. A. 2012. Improving Bayesian Population Dynamics Inference: A Coalescent-Based Model for Multiple Loci. *Molecular Biology and Evolution*, 30(3): 713–724.

[4] Heled, J. and Drummond, A. J. 2008. Bayesian inference of population size history from multiple loci. *BMC Evolutionary Biology*, 8(1): 289.

[5] Höhna, S., Landis, M. J., and Huelsenbeck, J. P. 2021. Parallel power posterior analyses for fast computation of marginal likelihoods in phylogenetics. *PeerJ*, 9: e12438.

[6] Minin, V. N., Bloomquist, E. W., and Suchard, M. A. 2008. Smooth Skyride through a Rough Skyline: Bayesian Coalescent-Based Inference of Population Dynamics. *Molecular Biology and Evolution*, 25(7): 1459–1471.

[7] Opgen-Rhein, R., Fahrmeir, L., and Strimmer, K. 2005. Inference of demographic history from genealogical trees using reversible jump markov chain monte carlo. *BMC Evolutionary Biology*, 5(1): 6.

[8] Vershinina, A. O., Heintzman, P. D., Froese, D. G., Zazula, G., Cassatt-Johnstone, M., Daln, L., Der Sarkissian, C., Dunn, S. G., Ermini, L., Gamba, C., Groves, P., Kapp, J. D., Mann, D. H., Seguin-Orlando, A., Southon, J., Stiller, M., Wooller, M. J., Baryshnikov, G., Gimranov, D., Scott, E., Hall, E., Hewitson, S., Kirillova, I., Kosintsev, P., Shidlovsky, F., Tong, H.-W., Tiunov, M. P., Vartanyan, S., Orlando, L., Corbett-Detig, R., MacPhee, R. D., and Shapiro, B. 2021. Ancient horse genomes reveal the timing and extent of dispersals across the bering land bridge. *Molecular Ecology*, 30(23): 6144–6161.

[9] Xie, W., Lewis, P. O., Fan, Y., Kuo, L., and Chen, M. H. 2011. Improving marginal likelihood estimation for Bayesian phylogenetic model selection. 60(2): 150–160.
